# An ensemble approach improves the prediction of the COVID-19 pandemic in South Korea

**DOI:** 10.7189/jogh.15.04079

**Published:** 2025-03-28

**Authors:** Kyulhee Han, Catherine Apio, Hanbyul Song, Bogyeom Lee, Xuwen Hu, Jiwon Park, Liu Zhe, Taewan Goo, Taesung Park

**Affiliations:** 1 Interdisciplinary Program of Bioinformatics, Seoul National University, Seoul, Korea; 2 Department of Industrial Engineering, Seoul National University, Seoul, Korea; 3 Department of Statistics, Seoul National University, Seoul, Korea

## Abstract

**Background:**

Modelling can contribute to disease prevention and control strategies. Accurate predictions of future cases and mortality rates were essential for establishing appropriate policies during the COVID-19 pandemic. However, no single model yielded definite conclusions, with each having specific strengths and weaknesses. Here we propose an ensemble learning approach which can offset the limitations of each model and improve prediction performances.

**Methods:**

We generated predictions for the transmission and impact of COVID-19 in South Korea using seven individual models, including mathematical, statistical, and machine learning approaches. We integrated these predictions using three ensemble methods: stacking, average, and weighted average ensemble (WAE). We used train and test errors to measure a model's performance and selected the best covariate combinations based on the lowest train error. We then evaluated model performance using five error measures (*r*^2^, weighted mean absolute percentage error (WMAPE), autoregressive integrated moving average (ARIMA), mean squared error (MSE), root mean squared error (RMSE), and mean absolute percentage error (MAPE)) and selected the optimal covariate combination accordingly. To validate the generalisability of our approach, we applied the same modelling framework to USA data.

**Results:**

Booster shot rate + Omicron variant BA.5 rate was the most commonly selected combination of covariates. For raw data evaluated using the WMAPE, individual models achieved the following: Generalised additive modelling (GAM) reached a value of 0.244 for the daily number of confirmed cases, a value of 0.172 for the time series Poisson for the daily number of confirmed deaths, and a value of 0.022 for both ARIMA and time series Poisson for the daily number of ICU patients. For smoothed data, the Holt-Winters model achieved a value of 0.058 for daily confirmed cases, while ARIMA attained a value of 0.058 for the daily number of confirmed deaths and 0.013 for the daily number of ICU patients. Among ensemble models, the SVM-based stacking ensemble achieved error values of 0.235 for the daily number of confirmed cases, 0.118 for the daily number of deaths, and 0.019 for the daily number of ICU patients on raw data. For smoothed data, the average ensemble and weighted average ensemble achieved 0.060 for the daily number of confirmed cases and 0.013 for daily ICU patients. The ensemble models also generalised well when applied to data from the USA.

Booster shot rate + Omicron variant BA.5 rate was the most commonly selected combination of covariates. For raw data, GAM (0.244) predicted daily confirmed cases best, time series Poisson (0.172) predicted daily confirmed deaths, and both ARIMA and time series Poisson (0.022) predicted daily ICU patients, based on WMAPE. For smoothed data, time series Poisson predicted daily confirmed cases (0.065) best, while ARIMA best predicted daily confirmed deaths (0.058) and ICU patients (0.013). For ensemble models, stacking ensemble using SVM was the best model for predicting daily confirmed cases (0.228), deaths (0.11), and ICU patients (0.02). With smoothed data, average ensemble and WAE were the best models for predicting daily confirmed cases (0.058) and ICU patients (0.011). The performance of ensemble models was generalised to other countries using the USA data for predictive performance.

**Conclusions:**

No single model performed consistently. While the ensemble models did not always provide the best predictions, a comparison of first-best and second-best models showed that they performed considerably better than the single models. If an ensemble model was not the best performing model, its performance was always not far from the best single model: a look at the mean and variance of the error measures shows that ensemble models provided stable predictions without much variation in their performances compared to single models. These results can be used to inform policymaking during future pandemics.

Every infectious disease outbreak exhibits specific spread dynamics that need to be identified based on the transmission patterns of a given disease outbreak [1]. With every epidemic and pandemic, the biggest challenge is the perception of the outbreak by the population: governments focus on the intervention measures to be taken, while the general population fears the impact of the outbreak on their health and everyday life. However, government intervention policies aimed at mitigating and suppressing the spread of the disease rely on the methods used to evaluate and forecast diseases when they occur [1]. Since 2019, the world has been dealing with COVID-19, a disease caused by SARS-CoV-2, a virus that spread from Wuhan, China, and led to a serious global public health crisis. Despite the preventive and control measures implemented in many countries, this novel coronavirus proved very contagious and spread globally, resulting in millions of cases [2]. Governments thus had to rely on the forecasting done by statistical, mathematical, and machine learning models to understand the situation, evaluate the severity of the pandemic, and establish well-tailored strategies and decisions to contain the disease and limit new infections [2].

In this context, the accuracy of traditional forecasting largely depends on available data to base its predictions and estimate uncertainty [3]. However, this data can vary in reliability due to the possibility of underreporting of the number of cases and deaths. A further challenge comes from the non-linear transmission patterns exhibited in most pandemics or epidemics [4]. With this in mind, various models have been proposed to predict the transmission of COVID-19. They are mainly grouped into statistical, mathematical and machine learning (or deep learning) models, which consider many response variables like the daily number of deaths, confirmed cases, intensive care unit (ICU) patients, and predictors like movement of individuals, the environment, and vaccination. However, each of these models has its advantages and shortcomings. Statistical models are mainly based on regression approaches; they are simple and straightforward [5], but their results are not indicative of a causal relationship in the data [6]. Time series models include autoregressive integrated moving averages (ARIMA), moving averages (MA), and auto-regressive (AR) methods. These approaches overwhelmingly depend on assumptions and underperform in forecasting real-time transmission rates [1]. Machine learning (or deep learning) models like the long short-term memory (LSTM) and bidirectional long short-term memory (Bi-LSTM) can capture both linear and nonlinear patterns in the data. However, they often operate as black boxes, are prone to overtraining and overfitting, require careful hyperparameter tuning, and demand a large amount of data for accurate forecasting [7]. Mathematical models, meanwhile, mainly involved the traditional compartmental susceptible, infected, and recovered (SIR) model and its variants; they, however, are known to have poor generalisation ability [8].

Despite these shortcomings, these models have largely been successful in their predictive capability and in informing government policies [3]. However, they provide different prediction results due to these shortcomings, which is why no single model can be used to make definite conclusions [9,10].

Therefore, there is an urgent need to validate the wide range of existing COVID-19 models to align their predictions, increase their transparency, and reduce individual model biases [11]. As a state-of-the-art solution for machine learning and deep learning challenges, ensemble learning has been highly recommended for achieving this purpose, as it often outperforms any single model by offsetting component biases due to reduced error variance [11-13]. In terms of function, ensemble learning combines predictions from multiple models known as components [12], which can be mathematical, curve-fitting, or agent-based models. It is broadly categorised into bagging, boosting, stacking, negative correlation-based deep ensemble models, explicit or implicit ensembles, homogeneous or heterogeneous ensembles, and decision fusion strategies-based deep ensemble models [13]. The ensemble components can also be combined using various algorithms, with the most common one being stacked generalisation (or simply stacking). One study generated a single ensemble model by averaging predictions derived from equally weighted components [11]. Other ensembles can be generated using the median, the trimmed mean to exclude extreme predictions, voting, Bayesian model averaging, multiple linear regression, and principal component regression [14-17].

We reviewed both frequently and less frequently used models in the forecasting of the COVID-19 pandemic and selected those that showed good performance. Some were shown to perform well in one of our previous works [18]. These methods include machine learning methods (Bi-LSTM and the light gradient-boosting machine (LightGBM)), statistical models (generalised additive modelling (GAM), ARIMA, Holt-Winters, and time series Poisson), and mathematical models (extended susceptible, exposed, infected, recovered, and deaths (SEIRD)). Each method was used to forecast COVID-19 daily number of confirmed cases, deaths, and ICU patients in South Korea [18]. We thus used an ensemble model compiled through stacking, averaging and weighted averaging ensemble of the seven single models to overcome each model’s limitations to better predict COVID-19 daily cases, deaths, and ICU patient situations in South Korea.

## METHODS

### Data collection

Our data consisted of a daily series of confirmed cases, death cases, ICU patients, vaccination rate (VR) per hundred people (based on the number of inoculations), and the stringency index (SI) for Korea, as retrieved from the Our World in Data website [19]. Specifically, we obtained the daily number of confirmed cases and deaths through the Center for Systems Science and Engineering at Johns Hopkins University [20], the SI from the Oxford COVID-19 Government Response Tracker [21], and the ICU patient and VR data from Our World in Data [22]. We supplemented the missing data on the daily number of confirmed cases, deaths, and ICU patients from Our World in Data on a specific date using Korea's COVID-19 dashboard [23]. Lastly, we retrieved the proportion of sequences by variant (including Omicron) from the CoVariants website [24] and the Global Initiative on Sharing Avian Influenza Data (GISAID) [25-27]. For the validation analysis using USA data, we collected the data in a similar manner as for data were collected in a similar manner as for Korea data was collected. No missing data were encountered, so we did not need to use additional sources such as Korea's COVID-19 dashboard.

### Data preparation

We used data from 1 January 2022 to 21 September 2022 in our analysis, setting the training period from January 1 to 14 September 2022 and using the last seven days (15 to 21 September 2022) as test data. To compensate for missing data, we first treated missing values preceding the first observation of each variable as 0. Then, we imputed the missing values after an observed value using the last observation carried forward (LOCF) method, replacing it at a later point as the last non-missing observation [28]. Lastly, we used linear interpolation to fill in a missing value that existed between actual observations. Because we only have the missing values preceding the first observation for the response variables, we only applied LOCF and linear interpolation for the covariates. Although we had the covariates for the test period, it is reasonable to assume the absence of covariates in an actual test scenario. Therefore, we generated a simulated dataset based on a simple scenario. For the test period from T + 1 to T + 7, we generated the covariates X_T + 1_, …, X_T + 7_ as follows:

X_T + 1_ = X_T − 7_ + (X_T − 7_ − X_T − 14_)

X_T + 7_ = X_T − 1_ + (X_T − 1_ − X_T − 8_)

This implies that during the test period, the change in covariates mirrored the change observed in the covariates from the previous week.

We also used both raw and smoothed data to capture changing trends and obtain higher prediction accuracy. Smoothed data referred to a seven-day simple moving average (SMA), since weekly patterns were observed in the confirmed cases. This may be due to variations of COVID-19 testing across the week, where fewer people are tested on weekends than on weekdays. Using raw and smoothed data might have led to differences in parameters for each model, differences in predictions for each country, and differences in forecasting accuracies, as several studies found that prediction errors were smaller when using smoothed data compared to raw data [29,30]. However, raw data provides predictive information closer to the real-world scenario (*e.g.* the maximum confirmed cases that will occur) compared to smoothed data. Since both have some implications for predicting future disease patterns, we used both raw and smoothed data were used for forecasting. The processed data used in our study is available in the dataset accompanying this article (**Online Supplementary Document**).

For the validation analysis using USA data, similar preparation steps were applied, except for the analysis period. We used data from 1 November 2021 to 28 February 2022, with the last seven days designated as test data. Missing value imputation, test data generation, and smoothing were all performed in the same manner described for the Korean data.

### Lagging

We aimed to predict the response variable *Y_t_*, the count time series at time *t*. Both smoothed and raw counts of new cases, deaths, and ICU patients were considered response variables. The covariates *X_t_* = (*x_1t_,…,x_Kt_*) included booster shot rate, rate of omicron subvariant BA.5, and SI. However, the impact of vaccinations and government intervention policies on the spread of COVID-19 may have taken some time before their effects were observed. Therefore, it would be reasonable to consider the time lapse between policy introduction and the observation of its impact when forecasting future the number of daily confirmed cases, daily death cases, or ICU patients. We thus considered four lag periods (seven, 14, 21, and 28 days) for VRs and SIs in our models. According to the World Health Organization [31], the incubation period for the SARS-COV-2 virus is around 5–6 days. Therefore, even if restriction policies are implemented, a minimum of seven days would be needed for its impact to be observed, which led us to choose seven as the minimum time lag. We further considered three additional lag periods to account for weekly effects, with a maximum duration of four weeks, as discussed in our previous paper [18].

### Models

Seven models were used to forecast the COVID-19 situation (daily cases, deaths, and ICU patients) in South Korea: time series autoregressive integrated moving average (ARIMA), extended SEIRD, time series Poisson, bidirectional long short-term memory (Bi-LSTM), generalised additive model (GAM), Holt-Winters, and light gradient-boosting machine (LightGBM) models (Figure S1 and Tables S1–4 in the **Online Supplementary Document**). The extended SEIRD we used here is a refined version of the SEIRD model that accounts for waning immunity over time. The multiplicative seasonal ARIMA models are fundamental for time series data, since seasonality can easily be applied to them, while the method also best reflects current observations that are greatly affected by observations of the day before than any other method [32]. The time series Poisson is another representative model for analysing data when current observations depend on past data; it uses previous conditional means in its prediction process, providing reasonable predictions [33]. The GAM model can deal with nonlinear relationships between predictors and response variables [34]. LightGBM, meanwhile, uses a leafwise tree growth boosting algorithm and continuously updates its tree structure, yielding good prediction performance in most cases [35]. The Bi-LSTM learns time series data bi-directionally, enhancing its ability to predict future data [36]. The Holt-Winters method is one of the basic time series prediction methods for forecasting, while accounting for trends and seasonality [37].

Many error measures can be used to assess accuracy, such as mean squared error (MSE), root mean squared error (RMSE), and mean absolute error (MAE). However, these measures have specific drawbacks. For example, MSE and MAE are scale-dependent, making them unsuitable for analysing the prediction performance of different response variables. Other error measures include mean absolute percentage error (MAPE), *r*^2^, and weighted mean absolute percentage error (WMAPE). Unlike MSE and MAE, MAPE is a scale-free measure, but suffers from singularity problems when the denominator is zero or exaggerates the error if the denominator is too small. WMAPE is the weighted version of MAPE. Therefore, we chose five error measures for our study analysis – MSE, RMSE, MAPE, *r*^2^ and WMAPE – to measure the forecasting accuracies of the selected methods. We defined them for both train and test data as follows:



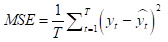





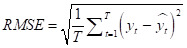





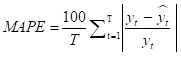





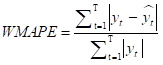





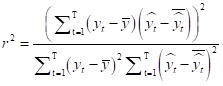



where *y_t_* and *y hat t* are actual and forecast values, respectively. The smaller the error value (or the larger *r*^2^), the better the model performance in predicting the response variable.

### Covariate combination selection

For each prediction model, we used different covariate combinations (booster shot rate (BSR), rate of omicron sub-variant BA.5 (BA.5 rate), and SI), along with various lag periods when fitting each dependent variable (new cases, deaths, and ICU patients for both smoothed and raw data). The null model (NULL) has only days as the explanatory variable in the model. The covariate combinations included BA.5 rate + SI, BSR + SI, and SI + BA.5 rate + BSR. Each error measure selected the covariate combination with the smallest train error. The final best covariate combination for a given response variable and model was selected if it was chosen by all error measures or the most common covariate combination picked by error measures. However, if the NULL model is selected with any covariate combination in equal numbers, the model with covariates is chosen over the NULL model with covariates. Unlike statistical and deep learning models, the extended SEIRD and the Holt-Winters models would require modification of their model structure to include the covariates, and are thus not considered in the best covariate combination analysis. The predicted results were used as inputs to the ensemble model. For the validation analysis using USA data, we followed an almost identical procedure as previously mentioned. However, the only difference was the usage of the Omicron rate instead of the BA.5 rate.

### Ensemble method

As we outlined previously, ensemble learning has been applied to various fields to improve prediction performance and reduce generalisation errors and bias by supplementing the limitation of each model [11-13]. We tried several ensemble methods to improve the performance of individual forecasting models and provide more stable predictions by using the best prediction result for each model. We used three types of ensemble approaches, including average ensemble, weighted average ensemble (WAE), and stacking ensemble using support vector machine (SVM) and multiple linear regression (LR).

### Average ensemble and WAE

First, an average ensemble is computed by simply taking the average of the prediction results of the individual models at a given time point. Second, a WAE is computed by taking the weighted average of the prediction results of the individual models at a given time point. There are various ways of defining weights for the prediction value of each model. Here, we assigned more weights to the prediction results from the better model. We used the normalised inverse of the training error values from the four error measures (MSE, RMSE, MAPE, and WMAPE) for each model and predictor variable to define weights for the WAE model. To determine the best weight for the WAE model for each predictor variable, the prediction performance of the WAE model trained with weights from each error measure value of each method was again measured using the four error measures and the most common error measure weight was selected as the best weight for the WAE model for a given predictor variable.

### Stacking ensemble

A stacking ensemble is an ensemble approach that uses the prediction results of the individual models as training data for another model. Here we used the prediction results of the training period to train the ensemble model. After training, we used the test results from the individual models to calculate the test results for the ensemble model. We used multiple LR and SVM as ensemble models in this study. Multiple LR is one of the simplest methods as an ensemble model, while SVM has the advantage of modelling a nonlinear relationship between results from each model and the actual response variable. In the training step, an ensemble model is fitted using the best prediction results of each model, (*e.g.* multiple LR and SVM). The prediction results become the covariates, while daily cases, ICU patients, and daily deaths are the response variables. We can obtain ensemble prediction results from the fitted ensemble models for both train and test periods. We fitted multiple LR using the ordinary least squares method; for the SVM, we used the epsilon regression with radial kernel. We developed all ensemble models in *R*, version 4.2.1 (R Core Team, Vienna, Austria), and used its ‘e1071’ package, version 1.7.11, for stacking the ensemble with SVM, and t na.fill() function and the na.approx() function from the ‘zoo’ package when addressing missing values.

## RESULTS

### Individual models

**Figure 1:**
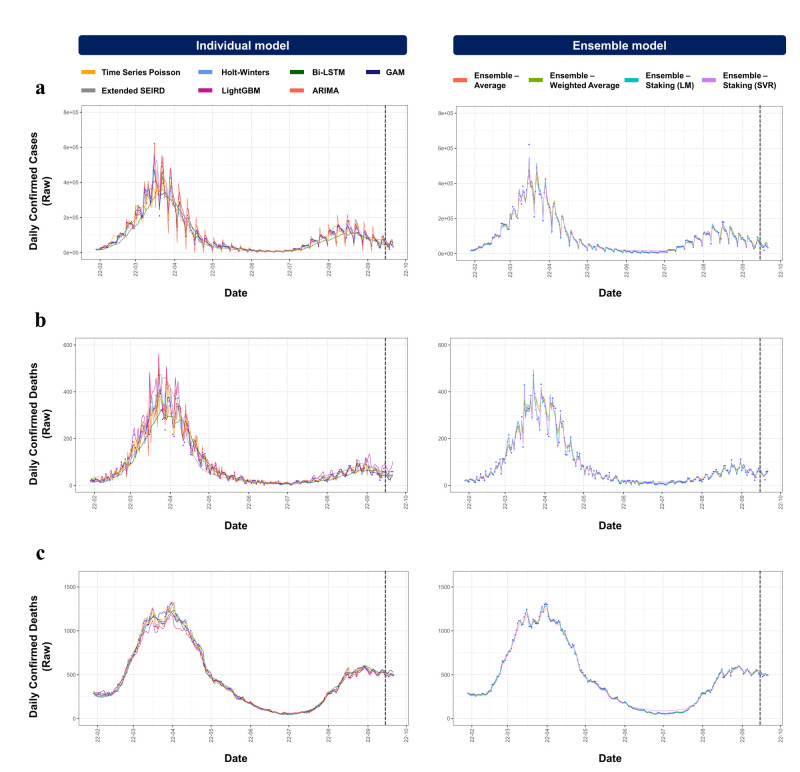
Plots showing the forecasting of the daily number of confirmed cases, confirmed deaths, and ICU patients using the seven individual models (ARIMA, GAM, LightGBM, Bi-LSTM, extended SIERD, and time series Poisson) and ensemble models with raw data. The right side of the vertical line marks the test period. ARIMA – autoregressive integrated moving average, GAM – generalised additive model, LightGBM – light gradient boosting machine, Bi-LSTM – bidirectional long short-term memory, extended SIERD – extended susceptible, infected, exposed, recovered, and deceased, LM – linear regression model, SVR – support vector regression.

The best covariate combination with their error value was selected based on training error for five out of seven methods (ARIMA, GAM, LightGBM, time series Poisson and Bi-LSTM) with each error measure method. Overall, the optimal covariate combination varied slightly depending on the error metric used, reflecting inherent differences in model performance across evaluation criteria (**Table 1**; Table S5–9 in the **Online Supplementary Document**). The GAM model selected the same set of covariate combinations across all response variables for both smoothed and raw data. The LightGBM selected BSR + BA.5 rate for both raw and smoothed ICU patients. Other methods selected different covariate combinations across all response variables with BSR  + BA.5 rate followed by NULL being prominent in the analysis (**Table 2**). Most individual models can forecast the overall pattern for both raw (**Figure 1**) and smoothed (Figure S2 in the **Online Supplementary Document**) data.

**Table 1:** The error values for the best covariate combination for each model and error method

	Daily number of confirmed cases (raw)					Daily number of confirmed cases (smoothed)				
	**MSE**	**RMSE**	**MAPE**	**WMAPE**	***r^2^***	**MSE**	**RMSE**	**MAPE**	**WMAPE**	***r^2^***
**ARIMA**	Null (8.381e +08)	Null (28950.097)	BA.5 rate (22.086)	BA.5 rate (0.167)	Null (0.941)	Null (2.62e +07)	Null (5118.226)	Null (4.503)	Null (0.037)	Null (0.998)
**Bi-LSTM**	Null (1.664e +09)	Null (40786.851)	BSR (64.057)	BSR (0.260)	Null (0.876)	BSR-BA.5 rate (1.545e +08)	BSR-BA.5 rate (12428.733)	BA.5 rate (41.062)	BSR-BA.5 rate (0.102)	BSR (0.998)
**GAM**	BSR-BA.5 rate (8.683e +08)	BSR-BA.5 rate (29467.697)	BSR-BA.5 rate (62.104)	BSR-BA.5 rate (0.215)	BSR-BA.5 rate (0.978)	BSR-BA.5 rate (6.49e +08)	BSR-BA.5 rate (25475.113)	BSR-BA.5 rate (64.910)	BSR-BA.5 rate (0.215)	BSR-BA.5 rate (0.996)
**LightGBM**	Null (4.517e +09)	Null (67211.360)	Null (54.702)	Null (0.432)	Null (0.986)	BA.5 rate (4.276e +09)	BA.5 rate (65390.524)	BA.5 rate (24.957)	BA.5 rate (0.381)	BA.5 rate (0.998)
**Time series Poisson**	BSR-BA.5 rate (2.489e +09)	BSR-BA.5 rate (49886.255)	BA.5 rate (51.036)	BSR-BA.5 rate (0.302)	BSR-BA.5 rate (0.914)	BSR-BA.5 rate (6.117e +08)	BSR-BA.5 rate (24732.946)	BSR-BA.5 rate (28.834)	BSR-BA.5 rate (0.172)	BSR-BA.5 rate (0.998)
	**Daily number of confirmed deaths (raw)**					**Daily number of confirmed deaths (smoothed)**				
	**MSE**	**RMSE**	**MAPE**	**WMAPE**	***r^2^***	**MSE**	**RMSE**	**MAPE**	**WMAPE**	***r^2^***
**ARIMA**	Null (4.132e +02)	Null (20.328)	Null (20.605)	Null (0.138)	Null (0.965)	Null (1.769e +01)	Null (4.206)	Null (3.864)	Null (0.032)	Null (0.998)
**Bi-LSTM**	Null (1.617e +03)	Null (40.211)	Null (61.811)	Null (0.300)	BSR (0.892)	BSR (2.408e +02)	BSR (15.519)	BSR (18.371)	BSR (0.110)	BSR (0.997)
**GAM**	BSR-BA.5 rate (2.195e +03)	BSR-BA.5 rate (46.852)	BSR-BA.5 rate (75.098)	BSR-BA.5 rate (0.393)	BSR-BA.5 rate (0.956)	BSR-BA.5 rate (2.024e +03)	BSR-BA.5 rate (44.989)	BSR-BA.5 rate (66.200)	BSR-BA.5 rate (0.406)	BSR-BA.5 rate (0.996)
**LightGBM**	BSR (2.433e +03)	BSR (49.329)	BSR (37.611)	BSR (0.348)	BSR-BA.5 rate (0.989)	BSR (1.682e +02)	BSR (12.969)	BSR-BA.5 rate (6.721)	BSR (0.076)	BA.5 rate (0.999)
**Time series Poisson**	BA.5 rate (2.073e +03)	BA.5 rate (45.527)	BA.5 rate (55.871)	BA.5 rate (0.312)	BA.5 rate (0.919)	Null (5.651e +02)	Null (23.772)	Null (25.893)	Null (0.164)	Null (0.998)
	**Daily number of ICU patients (raw)**					**Daily number of ICU patients (smoothed)**				
	**MSE**	**RMSE**	**MAPE**	**WMAPE**	***r^2^***	**MSE**	**RMSE**	**MAPE**	**WMAPE**	***r^2^***
**ARIMA**	BSR (2.217e +02)	BSR (14.891)	BSR (3.048)	BSR (0.024)	BSR (0.999)	BA.5 rate (3.335e +02)	BA.5 rate (18.261)	BA.5 rate (3.433)	BSR-BA.5 rate (0.029)	BA.5 rate (0.997)
**Bi-LSTM**	BSR-BA.5 rate (3.025e +03)	BSR-BA.5 rate (55.001)	BSR-BA.5 rate (15.106)	BSR-BA.5 rate (0.088)	BSR-BA.5 rate (0.990)	BSR (1.453e +03)	BSR (38.119)	Null (8.685)	BSR (0.054)	Null (0.998)
**GAM**	BSR-BA.5 rate (5.146e +04)	BSR-BA.5 rate (226.859)	BSR-BA.5 rate (58.032)	BSR-BA.5 rate (0.379)	BSR-BA.5 rate (0.991)	BSR-BA.5 rate (4.915e +04)	BSR-BA.5 rate (221.705)	BSR-BA.5 rate (52.757)	BSR-BA.5 rate (0.363)	BSR-BA.5 rate (0.994)
**LightGBM**	BSR-BA.5 rate (2.8e +03)	BSR-BA.5 rate (52.917)	BSR-BA.5 rate (5.772)	BSR-BA.5 rate (0.066)	BSR-BA.5 rate (0.996)	BSR-BA.5 rate (1.247e +03)	BSR-BA.5 rate (35.308)	BSR-BA.5 rate (8.269)	BSR-BA.5 rate (0.047)	BA.5 rate (0.998)
**Time series Poisson**	BSR (9.774e +03)	BSR (98.866)	BSR-BA.5 rate (24.373)	BSR-BA.5 rate (0.145)	BSR (0.993)	BSR-BA.5 rate (3.173e +04)	BSR-BA.5 rate (178.140)	BSR (52.489)	BSR-BA.5 rate (0.302)	BSR-BA.5 rate (0.999)

ARIMA – autoregressive integrated moving average, BA.5 rate – rate of omicron sub-variant BA.5, Bi-LSTM – bidirectional long short-term memory, BSR – booster shot rate, extended SIERD – extended susceptible, infected, exposed, recovered, and deceased, GAM – generalised additive model, LightGBM – light gradient boosting machine, LM – linear regression model, Null – The null model, SVR – support vector regression.

**Table 2:** Selected final best covariates combination for each method and predictor variable

	Daily number of confirmed cases (raw)	Daily number of confirmed cases (smoothed)
**ARIMA**	BA.5 rate	Null
**Bi-LSTM**	BSR	BSR + BA.5 rate
**GAM**	BSR + BA.5 rate	BSR + BA.5 rate
**LightGBM**	Null	BA.5 rate
**Time series Poisson**	BSR + BA.5 rate	BSR + BA.5 rate
	**Daily number of confirmed deaths (raw)**	**Daily number of confirmed deaths (smoothed)**
**ARIMA**	Null	Null
**Bi-LSTM**	Null	BSR
**GAM**	BSR + BA.5 rate	BSR + BA.5 rate
**LightGBM**	BSR	BSR
**Time series Poisson**	BA.5 rate	Null
	**Daily number of ICU patients (raw)**	**Daily number of ICU patients (smoothed)**
**ARIMA**	BSR	BA.5 rate
**Bi-LSTM**	BSR + BA.5 rate	BSR
**GAM**	BSR + BA.5 rate	BSR + BA.5 rate
**LightGBM**	BSR + BA.5 rate	BSR + BA.5 rate
**Time series Poisson**	BSR + BA.5 rate	BSR + BA.5 rate

ARIMA – autoregressive integrated moving average, BA.5 rate – rate of omicron sub-variant BA.5, Bi-LSTM – bidirectional long short-term memory, BSR – booster shot rate, extended SIERD – extended susceptible, infected, exposed, recovered, and deceased, GAM – generalised additive model, LightGBM – light gradient boosting machine, LM – linear regression model, Null – The null model, SVR – support vector regression.

**Figure 2:**
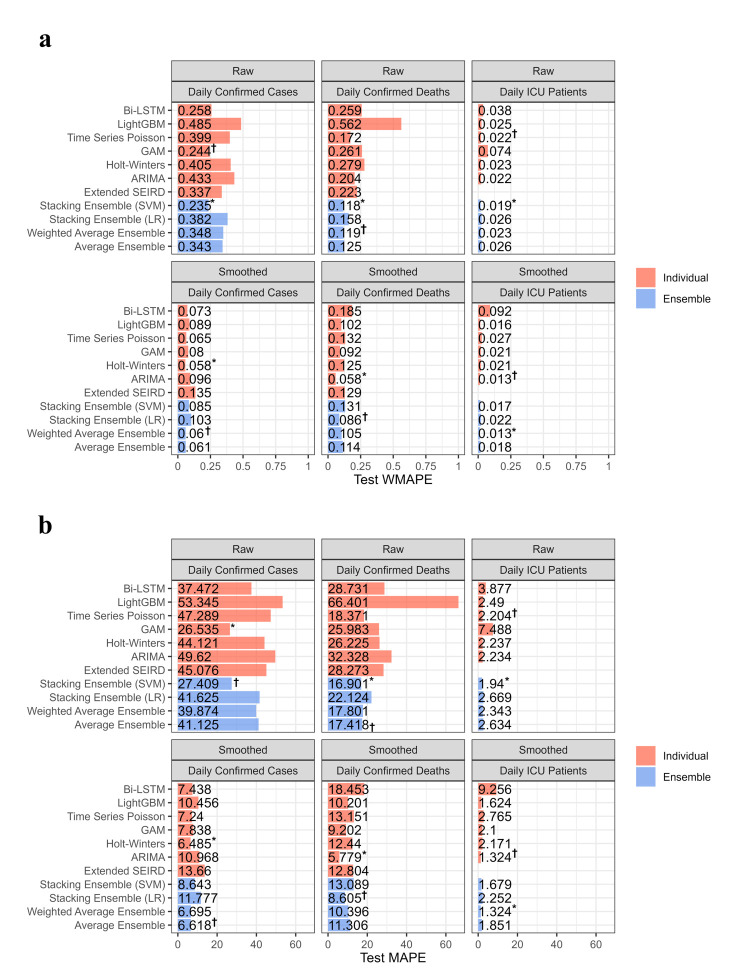
Summary of the performance of individual models and ensemble models using test data. **Panel A.** Performance using WMAPE values. **Panel B.** Performance using MAPE values. The horizontal bars represent the size of the error. The first-best performance is marked with * and the second-best performing model is marked in †. ARIMA – autoregressive integrated moving average, GAM – generalised additive model, LightGBM – light gradient boosting machine, Bi-LSTM – bidirectional long short-term memory, extended SIERD – extended susceptible, infected, exposed, recovered, and deceased, LM – linear regression model, SVR – support vector regression.

For the raw response variable, based on the WMAPE, the GAM achieved a value of 0.244 for the daily number of confirmed cases, time series Poisson recorded a value of 0.172 for the daily number of confirmed deaths, and both the ARIMA and the time series Poisson yielded values of 0.022 for the daily number of ICU patients (**Figure 2**). We observed a different pattern for smoothed response variable data. Holt-Winter’s (WMAPE = 0.058) was the best method for predicting the number of daily confirmed cases, while ARIMA was the best for the daily number of confirmed deaths (WMAPE = 0.058) and the daily number of ICU patients (WMAPE = 0.013). With MAPE and raw response variables, GAM (MAPE: 26.53) was the best model for predicting the daily number of confirmed cases and time series Poisson (MAPE = 18.37 and 2.20) for the daily number of confirmed deaths and the daily number of ICU patients, respectively. For smoothed response variables, Holt-Winter’s (MAPE = 6.49) was the best model for predicting the daily number of confirmed cases and ARIMA (MAPE = 5.78 and 1.32, respectively) for the daily number of confirmed deaths and the daily number of ICU patients, respectively. In summary, the performance of each model varies depending on the response variable predicted; therefore, no single model performs best across all response variables. The GAM models performed best for predicting the daily number of confirmed cases, the time series Poisson for the daily number of confirmed deaths, and Holt-Winter’s model for the daily number of ICU patients with raw data. The Holt-Winters model performed best in predicting the daily number of confirmed cases and ICU patients, while the ARIMA model worked best for predicting the daily number of confirmed deaths with smoothed data (Figures S3 and S4 in the **Online Supplementary Document**).

### Ensemble method

We fitted three ensemble models to combine the predictions of individual models. Specifically, for the WAE, five different error measures were considered, and the inverses of the MSE values were selected as weights in all WAE models, except for the smoothed daily number of confirmed ICU patients (Table S10 in the **Online Supplementary Document**). Ensemble models captured the overall pattern of epidemic spread more stably compared to individual models for both raw (**Figure 1**) and smoothed (Figure S2 in the **Online Supplementary Document**) data. Moreover, when comparing the performance of all models using WMAPE and MAPE values, ensemble models consistently showed more stable results (**Figure 2**). With WMAPE and raw response variables, stacking ensemble using SVM (values of 0.235, 0.118, and 0.019) was the best model for predicting the daily number of confirmed cases, deaths, and ICU patients, respectively. For the smoothed response variables, average ensemble and WAE (values of 0.06 and 0.013) were the best models for predicting the daily number of confirmed cases and ICU patients, respectively. Stacking an ensemble using LR (0.086) was the second-best method for predicting the daily number of confirmed deaths. With MAPE and raw response variables, a stacking ensemble using SVM (values of 27.4, 16.9, and 1.94) was the best model for predicting the daily number of confirmed cases, deaths, and ICU patients, respectively. For smoothed response variables, the average ensemble (value 6.6) was the best model for predicting the daily number of confirmed cases. Stacking ensemble using LR (value of 8.6) was the best model for predicting the daily number of confirmed deaths, and WAE (value of 1.32) for the daily number of ICU patients. Ensemble models gave stable predictions and capture the data patterns well.

### Overall model performances

To summarise the overall performance of all methods in this analysis for the best-performing models, we compared the first and second-best models across all outcome variables and the four error measures (Table S11 in the **Online Supplementary Document**). The first- and second-best models varied depending on the error measure used to measure the prediction performance of each model with each response variable, and depending on the use of smoothed and raw data. The error measures select different first-best and second-best models. For raw and smoothed response variables, out of the 30 best models, 18 and 13 were ensemble models. From this comparison, we observed that ensemble models were superior to the other models with raw data rather than with smoothed data.

### Validation analysis with USA data

**Figure 3:**
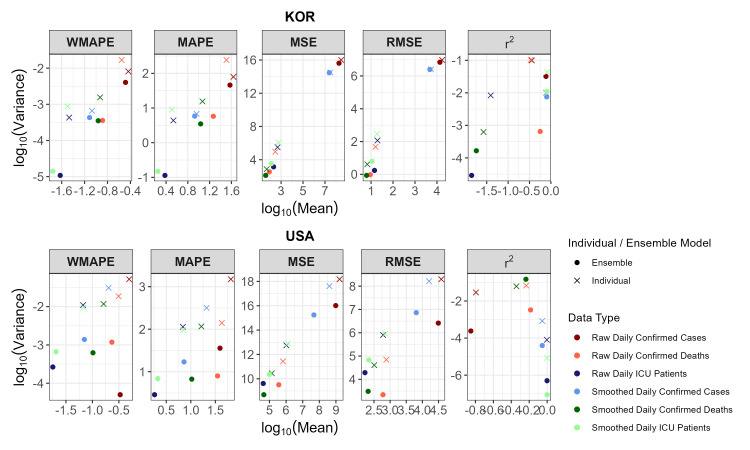
Mean and variance of error measures for Korea and USA analysis. The colours represent data type and the shapes represent individual and ensemble models.

For generalisability to other countries, we performed the same analysis using data from the USA. However, the analysis period and covariates differed from those used for the Korea data analysis, as we used data from 1 November 2021 to 21 February 2022 for training and data from 22 to 28 February 2022 (seven days) for testing. Covariate selection analysis was performed in the same manner as Korean data analysis (Table S12 in the **Online Supplementary Document**), and we fitted the three ensemble models to compare their performance to that of individual models (Figure S5 and Table S13 in the **Online Supplementary Document**). For both raw and smoothed response variables, out of the 30 first-best and second-best models, 13 and 21 were ensemble models, respectively. Here, ensemble models performed best with smoothed data, in contrast to the Korea analysis where ensemble models performed best with raw data. The USA raw data revealed extreme patterns with outliers and irregularities that could not be best captured by both the single and ensemble models. Smoothing of the data removed the outliers and irregularities from the data and improved the performance of both individual and ensemble models. We also observed that ensemble models give more stable predictive performances without many variations than the single models by comparing the variance and the log-transformed mean of all the error measures in USA and Korea analyses (**Figure 3**; Tables S14 and S15 in the **Online Supplementary Document**).

## DISCUSSION

Epidemiologists have used modelling to support a broad range of policy decisions, including during the COVID-19 pandemic [38], where it played an important role in predicting and controlling the spread of the disease. One of the most significant problems faced in that context is the development of models that can predict the evolution of the pandemic and estimate the effectiveness of various intervention measures and their impact on the economy [39]. This evidence would inform interventions that would reduce the burden the pandemic imposed on the healthcare system [40]. However, as these interventions came at a specific social cost [40], policymakers had to balancec intensity and timing. Forecasting results can be used for exactly this purpose. For example, based on prediction results of the number of COVID-19 ICU patients, the government can secure an appropriate number of beds; here, the more accurate the prediction results, the more precise the basis for this decision could be. More accurate predictions will create a more reliable foundation for decision-making. In practice, various models have been used to predict transmission trends for COVID-19, with their goal being to capture key factors that contribute to transmission [39].

However, the performances of these models often varied, as their inherent shortcomings impeded their accuracy. For example, machine learning models require a large amount of data and function as a black box, leading to overfitting and high computation-intensive hyperparameter tuning [41]. Mathematical models have been the backbone of epidemiological disease modeling. Their strengths come from their simplicity and robustness, even with insufficient data, such as in the early stages of an emerging infectious disease. However, they fail to capture the inherent heterogeneity of the underlying population, like mixing patterns within and across subpopulations due to mobility and failure to capture the effect of detailed intervention policies across communities [38]. Statistical models like time series Poisson, ARIMA, GAM, and others are data-dependent, making them unable to capture underlying causal mechanisms and, consequently, unable to capture epidemic dynamics that may arise due to behavioural adaptations [38].

Here we contrasted the performance of seven single models used in COVID-19 forecasting in Korea (ARIMA, GAM, extended SEIRD, LightGBM, time series Poisson, Holt-Winter’s, and Bi-LSTM models) using three response variables (daily confirmed cases, deaths, and ICU patients) and three covariates (SI, BA.5 rate, and BSR). Except for Holt-Winter’s and extended SEIRD models, we selected the best covariate combination with the smallest training error models. The BSR + BA.5 rate followed by NULL (no covariates added) were the most selected covariate combinations. The performance of the individual models was affected by whether the response variable was smooth or raw. Time series Poisson and ARIMA models stood out with consistently high prediction accuracies, especially for deaths and ICU patients. However, the lack of consistent performance from any model to predict all response variables well for both smoothed and raw data indicates that no single model could yield definite conclusions.

To overcome these shortcomings, research has suggested introducing modifications or other models, including ensemble modelling [42]. Ensemble approaches were mainly introduced to solve many deep learning challenges, but have also proven successful in fields such as time series forecasting and regression problems. In ensemble learning, many base classifiers are trained on a given dataset and their decisions are integrated into newer predictions [43]. This involves incorporating many individual models called ‘components’, an approach meant to overcome the shortcomings of a single model by coming up with a better, enhanced composite global model with higher accuracy and reliable estimates. For example, our analysis showed individual models may not capture the complex relationships with the actual response variables. The ARIMA model could handle seasonality and predicted peaks well, but also overemphasised weekly patterns. In contrast, Bi-LSTM followed the overall trend accurately, but failed to capture weekly patterns. Therefore, when the results of these models are combined and considered as inputs in the ensemble model, a better forecasting performance can be expected by offsetting the limitations of single model results and accurately predicting peaks while following the general pattern well. In our study, the stacking ensemble using SVM outperformed the other methods in forecasting the raw number of daily confirmed cases, deaths, and ICU patients.

Stacking is the most popular meta-learning integration technique, where the meta-learning model integrates the outputs of different base models built from different learning algorithms [13,44], and where the meta-model using a combiner algorithm then makes the final prediction [13,45]. The meta-learner tries to determine which base models are reliable or not [44]. The advantage of this approach lies in its exploiting of the independence between base learners. The errors made by one model differ from those found in another independent model, allowing the ensemble model to calculate the average of the errors [46]. In addition, stacking is a bias-reducing technique [47,48]. From our results, stacking an ensemble generally outperformed the other ensemble approaches and most single models in forecasting daily confirmed cases, deaths, and ICU patients. The good performance observed with the stacking ensemble (SVM) model can be attributed to the SVM’s ability to model nonlinear relationships among components, thus improving prediction.

Averaging ensemble approaches were mainly the second-best model after stacking ensemble, except for smooth data, where they sometimes emerged as the first-best model. Averaging is the most commonly used combination strategy for numerical outputs and includes simple and weighted averaging. Weighted averaging is fundamental in ensemble learning, since other combination methods are its variants. The combined output is the averaging of the outputs of the base learners with different weights for importance [49]. Typically, the weights are learned from training data; however, the learned weights are often unreliable due to data insufficiency or noise, especially in larger ensembles with many base learners due to overfitting. Further, many empirical studies and applications have shown that weighted averaging is not necessarily better than simple averaging [50,51]. Weighted averaging is therefore a better choice when individual learners have considerably different performances, while simple averaging is preferred when individual learners share comparable performances for the same task [44,52]. Other advantages of ensemble models include dealing well with small sample sizes, high dimensionality, reduced overfitting, bias or variance error, and recognition of data structural patterns in each component [53]. Its shortcomings include more complexity than single models and long computational time [54].

It is known, however, that the better prediction of ensemble learning also depends on the performance of the individual models. If a model performs well with the training data (due to overfitting) but fails to predict future trends, the ensemble models would produce less accurate results [55,56]. This may explain the better performance of some single models over ensemble models. From the results, we observed covariate combinations do not affect the performance of the ensemble or single models, since the covariate combination that provided the best prediction with each model was used. Smoothing of data has some impact. However, generally, the performances of single models were not consistent. Looking at the mean and variance of the error measures of ensemble models compared to the single models showed that ensemble models provide stable predictions without much variation in their performances compared to single models. By combining the results of single models, ensemble models overcome the limitations of single models to give predictions with reduced generalisation error and error variance, leading to more consistent results with fewer variations. Also, if the ensemble model is not the best performing model, its performance error measure is always not far from that of the best single model. The ensemble model’s accurate and stable predictions may assist policymakers in precisely understanding epidemic conditions and making reliable decisions.

Our study had some limitations that could have affected the accuracy of the models. First, many statistical methods exist for handling missing data, ranging from simple to model-based imputation methods. We applied the LOCF and linear interpolation methods for this purpose. These methods can alter the data distribution [57], introduce bias, and alter the patterns in time series data. Therefore, using different imputation methods could potentially affect the results. We performed a simple analysis to investigate the effects of imputation methods on the prediction performance of our results, where we fitted the ARIMA model to predict the daily raw confirmed cases using BA.5 rate data imputed by three different imputation methods (linear interpolation, LOCF, EWMA), subsequently comparing their results (Figure S6 in the **Online Supplementary Document**). There was no significant effect on prediction results when different imputation methods were used. The test WMAPE values were 0.6, 0.527 and 0.6 for linear interpolation, LOCF, and the exponentially weighted moving average, respectively.

Several analytical approaches can be considered in the future. First, ensemble analysis can be adapted for other diseases. Previous studies have conducted forecasting for influenza and dengue fever [58,59]. Each infectious disease has its specific characteristics – different vectors through which it is transmitted, different transmission rates, and different incubation periods – which require tailored modelling analysis. Detailed individual prediction models can be developed by considering different covariates for each disease, and the predictions from each model can be integrated using the ensemble models to provide more accurate, disease-specific forecasts. Second, while several methods provide different prediction results, no information is available on how reliable and precise the estimates are. Confidence intervals would provide such information about the uncertainty of predictions. However, no methodological framework for deriving confidence intervals of prediction values is currently available for ensemble and artificial intelligence/machine learning models, except for statistical models (Figure S7 in the **Online Supplementary Document**). Therefore, we are developing a bootstrap-based confidence interval calculation framework that can be applied to various models. This framework consists of two steps: generating block bootstrap data while considering the characteristics of time-series data and fitting individual models and ensemble models to the generated bootstrap data to calculate confidence intervals. We will present these findings in a future report.

## CONCLUSIONS

Many studies have been done on prediction models used in the forecasting of COVID-19 in different countries under different factors like mobility, intervention policies, SARS-COV-2 variants, and so forth. They have highlighted the shortcomings of these models and proposed various solutions. Here we considered seven models and different factors (covariates) that can affect COVID-19 forecasting, modelling different combinations of these factors and selecting the best combination that improved a model's performance. We found that no single model gave consistent performance across all response variables and, therefore, no single model gave definite conclusions. To address this, we used ensemble learning, whereby we developed a model that considered the prediction patterns presented by each of the single model components and combined them to achieve more accurate predictions. Our ensemble model produced more stable predictions, without the variations inherent to single models. These results can be used to inform policies and decision-making during future pandemics.

## Additional material


Online Supplementary Document
