# Supplementary material for: An ensemble approach improves the prediction of the COVID-19 pandemic in South Korea
Source: J Glob Health. 2025 Mar 28;15:04079. doi: 10.7189/jogh.15.04079 (PMC11949510; doi:10.7189/jogh.15.04079)
Supplement: Online Supplementary Document [file jogh-15-04079-s001.pdf]

Supplement to: Han K, Apio C, Song H, Lee B, Hu X, Park J, Zhe L, Goo T, Park T. An ensemble approach improves the prediction of the COVID-19 pandemic in South Korea. J Glob Health. 2025;15:04079.

Methods

1.1 Statistical models

1.1.1 AutoRegressive Integrated Moving Average

AutoRegressive Moving Average (ARMA) models for time series analysis were first suggested in Time Series Analysis: Forecasting and Control [1]. Since ARMA models could be applied only to stationary time series, AutoRegressive Integrated Moving Average (ARIMA) models utilized differentiation. Using the backshift operator B, we first define the following:

$$BY_t = Y_t - 1,$$

$$\varepsilon_t \sim \mathrm{WN}(0, \sigma^2),$$

$$\Phi(B) = 1 - \Phi_1 B - \Phi_2 B^2 - \cdots - \Phi_p B^p,$$

$\theta(B) = 1 + \theta_1 B + \theta_2 B^2 + \cdots + \theta_1 B^q$,

where X_t and ε_t refer to the real value and white noise error of a time series object t, at time respectively. The variance of white noise is given as σ^2. Then, a standard ARIMA model with orders p, d, and q is given as follows:

$$\Phi(B)(1 - B)^d = \theta(B)\varepsilon_t$$

Furthermore, multiplicative seasonal ARIMA models were developed to include seasonality in ARIMA models [2]. Given orders p, d, q, P, D, Q, and the span of seasonality s, the model is written as follows:

$$\Phi(B)\Phi(B^s)(1-B)^d(1-B^s)^D Y_t = \theta(B)\theta(B^s)\varepsilon_t$$

Finally, to obtain future predictions, both base ARIMA models and multiple regression with additional predictors and seasonal ARIMA errors were applied. The R package *forecast* was used for fitting ARIMA and seasonal ARIMA models. Meanwhile, the principle of parsimony was applied to this study. In contrast to previous studies [3] which simply utilized the *auto.arima()* function in R, our study compared Akaike Information Criterion (AIC) and Bayesian Information Criterion (BIC) values for all possible seasonal ARIMA models that fit and chose the best model by limiting the orders of models to integer values chosen beforehand. This helped to avoid overfitting issues.

1.1.2 Holt-Winters model

Holt-Winters Exponential Smoothing, also known as the Holt-Winters method, is a time series forecasting technique introduced by Holt and Winters for dealing with data with seasonality [4,5]. This model extends simple exponential smoothing by adding components for trend and seasonality, making it suitable for more complex time series data. The Holt-Winters method consists of three equations to update level ℓ_t, trend b_t, and seasonal s_t components:

$$\ell_t = \alpha(Y_t - s_{t-s}) + (1 - \alpha)(\ell_{t-1} + b_{t-1})\backslash$$

$$b_t = \beta(\ell_t - \ell_{t-1}) + (1 - \beta)b_{t-1}$$

$$s_t = \gamma(Y_t - \ell_t) + (1 - \gamma)s_{t-s}$$

Where, α, β, and γ are smoothing parameters for level, trend, and seasonality respectively, and s represents the length of the seasonality cycle.

Forecasting future values is performed using the formula:

$$\hat{Y}_{t+h} = \mathrm{l_t} + \mathrm{hb_t} + \mathrm{s_{t+h-s(k+1)}}$$

where h is the forecast horizon and k is the integer part of (h-1)/s.

The Holt-Winters model has two variations: additive and multiplicative, depending on the nature of the seasonal component. The additive model is used when seasonal variations are roughly constant through the series, while the multiplicative model is used when seasonal variations are proportional to the level of the series.

To apply this model in practice, we utilized the built-in function *HoltWinters()* in R. We assumed s = 7, indicating that seasonality repeats weekly. We selected the best model (additive or multiplicative) that provided the better training Weighted Mean Absolute Percentage Error (WMAPE).

1.1.3 Time series Poisson

The time series Poisson model is a form of time series model for count data fitted using the generalized linear models (GLM) for time series counts. Models for count time series should consider that the observations are nonnegative integers, and they should capture suitably the dependence among observations. A convenient and flexible approach is to employ the GLM methodology [6] for modeling the observations conditionally on past information. This methodology is implemented by choosing a suitable distribution for count data and an appropriate link function. Let F_t the history of the joint process $\{Y_t, \lambda_t, X_{t+1}\}$. We aim to model the conditional mean $E(Y_t|F_{t-1})$ by a process $\{\lambda_t\}$, such that $E(Y_t|F_{t-1}) = \lambda_t$. The general form of the model is given as follows:

$$g\,(\lambda_t) = \beta_0 + \sum_{m\in M}\beta_m\tilde{g}(Y_{t-m}) + \sum_{l\in L}\alpha_l g(\lambda_{t-l}) + \eta^t X_t \qquad (10)$$

where g and $\tilde{g}$ are the link and transformation functions, respectively. M and L are sets of natural numbers which determine the set of past observations or set of past linear predictors used to forecast current data. η represents the effects of covariates. In this study, to consider negative covariate effects, we used the logarithmic link function and let $g(x) = \log(x)\ and\ \tilde{g}(x) = \log(x+1)$. Then the above model can be written again as follows:

$$v_t = \beta_0 + \sum_{m=1}^{M} \beta_m \log(Y_{t-k} + 1) + \sum_{l=1}^{L} \alpha_l v_{t-l} + \eta^t X_t \quad (11)$$

where v_t is the linear predictor such that v_t = log (λ_t). We also applied the Poisson assumption for this model, i.e., $Y_t|F_{t-1}$ ~ Poisson (λ_t). tsglm Poisson models are introduced in the *tscount*: *An R Package for Analysis of Count Time Series Following Generalized Linear Models* (tsglm) [7].

1.1.4 Generalized Additive Model

The Generalized Additive Model (GAM) is a regression model that allows the learning of non-linear relationships between the predictors and response variables [8]. The model specifies the exponential family (binomial, normal, Poisson, etc.) for Y_t. The link function g is used to relate the expectation of Y_t to covariate vector X_t. For each covariate x_{kt}, GAM allows a non-linear relationship between each predictor and $\mathrm{E}(Y_t)$, using the smooth function $f_k(x_{kt})$. We can define the general form of this model as follows:

$$g(\mathrm{E}(Y_t)) = \beta_0 + f_1(x_{1t}) + \cdots + f_K(x_{Kt}),$$

when there are K covariates. After assuming Y_t ~ Poisson (λ_t), our model is as follows:

$$v_t = \beta_0 + \sum_{k=1}^{K} f_k(x_{kt})$$

where v_t is the linear predictor such that $v_t = \log(\lambda_t)$. Different smoothing functions f_k were used depending on the covariate. For weekdays and dates, cubic splines and P-splines were used, respectively. Thin plate regression splines were used for vaccination variables and SI [9]. R package *mgcv* was used for fitting GAM models [10,11].

1.2 Mathematical models

1.2.1 Extended SEIRD model

There are many classical susceptible-infectious-recovered models. Their popularity for modeling the spread of the pandemic is because of their simplicity. Here, we considered a susceptible-exposed-

infectious-recovered-death (SEIRD) mathematical model since in some epidemics, there exists a period when signs and symptoms are nonvisible or obvious, but infectious. In such a case, SEIRD is more realistic for considering the incubation time of the infectious disease. The SEIRD model consists of a system of non-linear ordinary differential equations to present the process of transmission. In this model, every individual either belongs to susceptible (S), exposed (E), infected (I), recovered (R) or Death (D). Thus, N = S + E + I + R + D represents the total population, which incorporates the COVID-19 vaccines' waning effect for improved predictions. To consider the waning of immunity after infection, we assumed that some of the recovered group may revert to the susceptible group and added the waning parameter ω. We referred to this model as extended SEIRD as shown in **Figure S1**. The waning effect refers to a decline in the level of immunity provided by a vaccine over time. This can occur due to a variety of factors such as the decline of antibody concentrations in the body, loss of immune memory, and the emergence of vaccine-resistant strains [12]. The waning effect can therefore lead to an increased susceptibility to infection and necessitates additional doses for adequate protection.

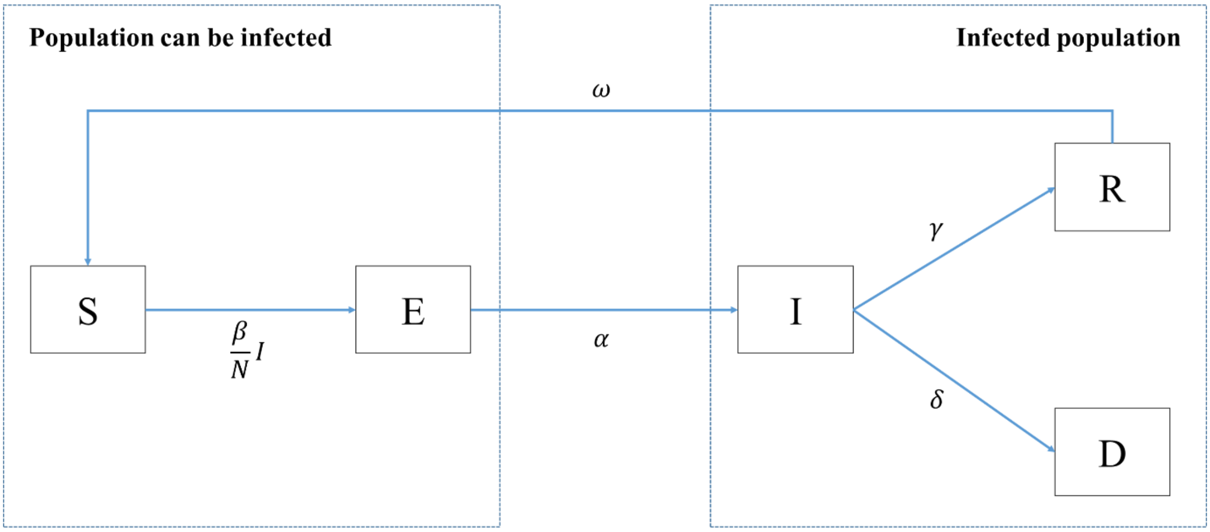

Figure S1. Structure of the extended SEIRD model.

Due to the variation and policy changes over time, the infection rate and the transmission rate also changed with time. Therefore, we divided the dataset into biweekly periods so that accurate parameters could be obtained. For every two weeks, we separately estimated the infection rate, transmission rate, and death rate parameters by using the sum of the square error to choose the optimal parameter value. On the other hand, possibly due to limited access to testing or reporting, the underreporting rate for infected cases was considered and set to 0.35 [13]. The parameters considered are summarized in **Table S1**.

Table S1. List of parameter values of the extended SEIRD model

Parameter symbol	Description	Value
α	infection (onset) rate	[1/4 ~ 1/1.5] (chosen by model)
β	transmission rate	[0.04 ~ 0.27] (chosen by model)
γ	recovery rate	1/7
δ	death rate	[1/800 ~ 1/1100] (chosen by model)
ω	waning effect	4/365
ρ	underreporting rate	0.35

1.3 Machine learning models

1.3.1 Light gradient-boosting machine

Light gradient-boosting machine (LightGBM) is a gradient-boosting decision tree algorithm that can be used for tasks like regression and classification. LightGBM is a histogram-based gradient boosting framework, so it has a faster training speed, lower memory usage and better accuracy than other boosting machines. As gradient boosting machines do, LightGBM consists of weak learners and decision trees. The decision tree uses the input data to determine which learner is the best to make predictions.

Based on the adaptive boosting algorithm, gradient boosting machines can build a strong regression learner by iteratively combining a set of weak regression learners. Gradient boosting

machines use gradient descent to minimize the loss function of a strong regression learner. Like other boosting algorithms, LightGBM adds models into the tree using greedy style [14];

$$F_m(X_t) = F_{m-1}(X_t) + \rho_m h_m(X_t)$$

where; F_m is the updated model, F_{m-1} is the previous model and $\rho_m h_m$ is the newly added model. h_m is the trained base learner which minimizes the loss function L, and ρ is the multiplier which is found by solving the one-dimensional optimization problem;

$$\rho_m = argmin[\Sigma \mathrm{L}(Y_t, \mathrm{F_{m-1}}(X_t) + \rho h_m(X_t)]$$

To build our LightGBM model, the 'LightGBM' package in Python was used [15]. We also employed the Jupyter Notebook and the *tmux* to run the model. The hyperparameters considered in the LightGBM model are summarized in **Table S2**. All options not listed in the table were set to their default values.

Table S2. The hyperparameter settings used in the LightGBM model.

Hyperparameter	Range	Explanation
**objective**	regression	The loss function for the model. Common choices include mean squared error for regression tasks and binary log loss for classification tasks.
**num_leaves**	[4, 8, 16, 32]	The number of leaf nodes in a tree.
**max_depth**	[3, 5,8, 10,16]	The maximum depth of a tree.
**min_split_gain**	0.001	The minimum gain required to split a node.
**min_child_samples**	[2, 4,8,16,32]	The minimum number of samples required in a leaf node.
**num_iterations**	2000	The number of iterations (or trees) in the model.
**early_stopping_round**	50	The number of rounds with no improvement on the validation set before training is stopped early.
**reg_alpha**	[0, 0.1, 0.3, 1]	L1 regularization coefficient.

**subsample_freq**	5	The frequency of subsampling the data.
**colsample_bytree**	0.6,0.8	The fraction of columns (features) to be sampled for each tree.
**bagging_seed**	777	The random seed used for subsampling or task distribution
**reg_lambda**	[0, 0.1, 0.3, 1]	L2 regularization coefficient
**learning_rate**	[0.1,0.01,0.05]	The "shrinkage parameter," controls how much the model's weights are updated in each iteration.
**boosting_type**	gbdt	Type of boosting to be used in the model.

1.3.2 Bidirectional Long Short-Term Memory Network

To deal with time series data, we considered a Long Short-Term Memory (LSTM) network as a deep learning approach [16]. Since LSTM takes only past information when training, we introduced Bidirectional LSTM (Bi-LSTM) to consider backward propagation information as well [17]. To get a better prediction, we used past observation as a covariate to forecast the Y_t. The optimal lag period is selected among 7, 14, or 21 which yields the least validation Mean Squared Error (MSE). Each block of LSTM computes the activation vector and the hidden state using calculation results from the previous block with the current input data X_t and past observations. The training process is conducted in both forward and backward directions to improve the model performance. Also, to decide the best model structure, we considered two hyperparameters: layer number {2, 3 and dropout rate {0, 0.2}. All the combinations of the hyperparameters were tested and one combination with the lowest validation MSE was selected. The model was developed in Python version 3.7.6 using Keras (Version 2.4.3, https://github.com/keras-team/keras) and TensorFlow (Version 2.3.0, https://github.com/tensorflow/tensorflow) libraries. We also employed the Jupyter Notebook and the *tmux* to run the model, and CUDA (Compute Unified Device Architecture) was used to accelerate the computation using the GPU. The hyperparameters considered in the Bi-LSTM model are summarized in **Table S3**. All options not listed in the table were set to their default values.

Table S3. The hyperparameter settings used in the Bi-LSTM model.

Hyperparameter	Range	Explanation
**Lag period**	[7, 14, 21, 28]	The number of past time steps used to predict the future step(s) in time series analysis.
**# of layer**	[2, 3, 4, 5]	The number of layers in a neural network.
**Dropout rate**	[0, 0.1, 0.2, 0.3]	The fraction of the input units dropped during training.
**Optimizer**	Adam	The algorithm is used to update the weights of the network during training.
**Activation function**	Hyperbolic Tangent	The non-linear transformation is applied to the input that decides whether a neuron should be activated or not.
**Loss function**	Mean Squared Error	The metric is used to measure the difference between the model's predictions and the actual data.

Table S4. The computational demand for each forecasting model. Please note that the table does not represent the minimum or recommended environment for each method but simply shows the approximate time required in a similar environment.

Method	Local/server	CPU (# Cores)	GPU (GRAM)	Main memory	Time	Language
Extended SEIRD	PC	1x Intel i5-1135G7 (8)	not used	32GB	~10 mins	R v4.2.3
tsglm	PC	1x Apple M2 Pro (12)	not used	32GB	~10 mins	R v4.2.3
ARIMA	PC	1x Intel i7-13700 (16)	not used	32GB	~10 mins	R v4.3.2
GAM	PC	1x Intel i7-13700 (16)	not used	32GB	~10 mins	R v4.3.2
LightGBM	GPU HPC	2x Intel Xeon(R) Gold 5220R (24)	1x NVIDIA TITAN RTX 24GB	93GB	~10 mins	python v3.9.13
Bi-LSTM	GPU HPC	2x Intel Xeon(R) Gold 5220R (24)	1x NVIDIA TITAN RTX 24GB	93GB	144 mins	python v3.7.6
Average ensemble	PC	1x Intel i7-13700 (16)	not used	32GB	~10 mins	R v4.3.2
Weighted average ensemble	PC	1x Intel i7-13700 (16)	not used	32GB	~10 mins	R v4.3.2
Stacking ensemble – LR	PC	1x Intel i7-13700 (16)	not used	32GB	~10 mins	R v4.3.2
Stacking ensemble – SVM	PC	1x Intel i7-13700 (16)	not used	32GB	~10 mins	R v4.3.2

Results

Table S5. The WMAPE values for all covariate combinations for each single model for Korea.

	Daily Confirmed Cases (Raw)							Daily Confirmed Cases (Smoothed)						
	ARIMA	Bi-LSTM	Extended SEIRD	GAM	Holt-Winters	LightGBM	Time Series Poisson	ARIMA	Bi-LSTM	Extended SEIRD	GAM	Holt-Winters	LightGBM	Time Series Poisson
**Null**	0.167	0.246	**0.185**	0.215	**0.140**	**0.108**	0.227	**0.037**	0.102	**0.104**	0.215	**0.022**	0.146	0.024
**BA.5 rate**	**0.166**	0.245		0.117		0.179	0.166	0.037	0.056		0.099		**0.090**	0.024
**BSR**	0.167	**0.243**		0.088		0.432	0.302	0.037	0.062		0.053		0.145	0.172
**BSR+BA.5 rate**	0.167	0.260		**0.086**		0.140	**0.161**	0.037	**0.051**		**0.046**		0.381	**0.023**

	Daily confirmed deaths (Raw)							Daily confirmed deaths (Smoothed)						
	ARIMA	Bi-LSTM	Extended SEIRD	GAM	Holt-Winters	LightGBM	Time Series Poisson	ARIMA	Bi-LSTM	Extended SEIRD	GAM	Holt-Winters	LightGBM	Time Series Poisson
**Null**	**0.129**	**0.250**	**0.274**	0.393	**0.214**	0.267	0.252	**0.031**	0.091	**0.173**	0.406	**0.039**	0.074	**0.033**
**BA.5 rate**	0.134	0.300		0.246		0.282	**0.196**	0.032	0.110		0.218		0.076	0.164
**BSR**	0.135	0.260		0.150		**0.171**	0.223	0.032	**0.060**		0.061		**0.073**	0.102
**BSR+BA.5 rate**	0.138	0.283		**0.148**		0.348	0.312	0.032	0.108		**0.050**		0.074	0.100

	ICU patients (Raw)							ICU patients (Smoothed)						
	ARIMA	Bi-LSTM	Extended SEIRD	GAM	Holt-Winters	LightGBM	Time Series Poisson	ARIMA	Bi-LSTM	Extended SEIRD	GAM	Holt-Winters	LightGBM	Time Series Poisson
**Null**	0.023	0.059		0.379	**0.044**	0.054	0.041	0.029	0.028		0.363	**0.012**	0.045	0.302
**BA.5 rate**	0.024	0.073		0.200		0.058	0.145	0.028	0.035		0.197		0.042	0.019
**BSR**	**0.020**	0.088		0.054		0.066	0.039	0.028	**0.028**		0.045		0.047	0.013
**BSR+BA.5 rate**	0.021	**0.052**		**0.045**		**0.053**	**0.039**	**0.028**	0.054		**0.039**		**0.034**	**0.013**

Table S6. The MAPE values for all covariate combinations for each single model for Korea.

	ARIMA	Bi-LSTM	Extended SEIRD	GAM	Holt-Winters	LightGBM	Time Series Poisson
**Daily Confirmed Cases (Raw)**							
**Null**	21.283	64.057	**25.426**	62.104	**12.845**	**13.251**	28.305
**BA.5 rate**	**21.267**	47.836		16.347		16.304	**20.186**
**BSR**	21.966	**34.880**		15.683		54.702	51.036
**BSR+BA.5 rate**	22.086	45.976		**13.330**		13.294	20.633
**Daily confirmed deaths (Raw)**							
**Null**	**17.977**	**32.922**	**39.571**	75.098	**30.755**	23.125	50.067
**BA.5 rate**	19.708	61.811		43.981		27.993	**35.319**
**BSR**	19.433	33.314		24.619		**22.389**	36.313
**BSR+BA.5 rate**	20.605	46.843		**23.667**		37.611	55.871
**ICU patients (Raw)**							
**Null**	2.806	8.180		58.032	**4.917**	5.772	4.814
**BA.5 rate**	3.048	9.810		27.983		5.490	24.373
**BSR**	**2.572**	15.106		8.250		5.720	4.485
**BSR+BA.5 rate**	2.787	**6.607**		**5.135**		**5.477**	**4.472**

	ARIMA	Bi-LSTM	Extended SEIRD	GAM	Holt-Winters	LightGBM	Time Series Poisson
**Daily Confirmed Cases (Smoothed)**							
**Null**	**4.313**	41.062	**14.571**	64.910	**2.366**	10.778	3.123
**BA.5 rate**	4.428	**18.447**		14.779		**5.710**	2.771
**BSR**	4.392	22.753		13.331		11.259	28.834
**BSR+BA.5 rate**	4.503	19.103		**8.041**		24.957	**2.743**
**Daily confirmed deaths (Smoothed)**							
**Null**	**3.731**	13.738	**26.018**	66.200	**6.553**	6.240	**5.027**
**BA.5 rate**	3.856	14.379		39.528		6.213	25.893
**BSR**	3.864	**8.478**		10.609		6.721	16.549
**BSR+BA.5 rate**	3.852	18.371		**8.809**		**6.014**	16.379
**ICU patients (Smoothed)**							
**Null**	3.433	**3.854**		52.757	**1.232**	8.269	52.489
**BA.5 rate**	**3.321**	5.129		27.281		5.471	2.494
**BSR**	3.328	3.980		7.306		4.682	**1.655**
**BSR+BA.5 rate**	3.327	8.685		**4.341**		**3.714**	1.658

Table S7. The RMSE values for all covariate combinations for each single model for Korea.

Daily Confirmed Cases (Raw)

	ARIMA	Bi-LSTM	Extended SEIRD	GAM	Holt-Winters	LightGBM	Time Series Poisson
**Null**	**28923.849**	**39415.273**	**31675.730**	29467.697	**28343.635**	**18306.154**	36377.318
**BA.5 rate**	28950.097	39755.564		21329.940		31419.255	33541.999
**BSR**	28930.803	40786.851		15970.159		67211.360	49886.255
**BSR+BA.5 rate**	28928.642	40064.247		**15812.395**		23820.078	**32033.322**

Daily Confirmed Cases (Smoothed)

	ARIMA	Bi-LSTM	Extended SEIRD	GAM	Holt-Winters	LightGBM	Time Series Poisson
**Null**	**5105.091**	12428.733	**13142.334**	25475.113	**4671.020**	23269.386	4959.369
**BA.5 rate**	5117.707	7787.977		15310.804		**15942.346**	4843.437
**BSR**	5111.678	7009.923		6850.230		23262.792	24732.946
**BSR+BA.5 rate**	5118.226	**6167.074**		**6731.694**		65390.524	**4495.094**

Daily confirmed deaths (Raw)

	ARIMA	Bi-LSTM	Extended SEIRD	GAM	Holt-Winters	LightGBM	Time Series Poisson
**Null**	**19.602**	**36.450**	**39.963**	46.852	**32.665**	43.678	36.206
**BA.5 rate**	20.200	40.211		32.153		49.329	**30.186**
**BSR**	20.189	36.519		21.842		**25.539**	35.657
**BSR+BA.5 rate**	20.328	37.663		**21.464**		46.860	45.527

Daily confirmed deaths (Smoothed)

	ARIMA	Bi-LSTM	Extended SEIRD	GAM	Holt-Winters	LightGBM	Time Series Poisson
**Null**	**4.188**	11.192	**23.865**	44.989	**5.251**	12.514	**4.766**
**BA.5 rate**	4.206	15.149		26.337		12.880	23.772
**BSR**	4.194	**7.461**		7.537		**12.280**	14.309
**BSR+BA.5 rate**	4.194	15.519		**6.261**		12.969	14.028

ICU patients (Raw)

	ARIMA	Bi-LSTM	Extended SEIRD	GAM	Holt-Winters	LightGBM	Time Series Poisson
**Null**	14.564	39.948		226.859	**32.469**	41.682	30.939
**BA.5 rate**	14.891	50.742		135.915		42.601	98.866
**BSR**	**12.583**	55.001		36.711		52.917	**29.153**
**BSR+BA.5 rate**	12.687	**34.607**		**33.684**		**40.990**	29.156

ICU patients (Smoothed)

	ARIMA	Bi-LSTM	Extended SEIRD	GAM	Holt-Winters	LightGBM	Time Series Poisson
**Null**	18.261	19.529		221.705	**11.186**	28.105	178.140
**BA.5 rate**	**17.638**	22.250		136.026		30.124	13.670
**BSR**	17.669	**18.786**		29.083		35.308	8.744
**BSR+BA.5 rate**	17.666	38.119		**28.267**		**25.756**	**8.715**

Table S8. The MSE values for all covariate combinations for each single model for Korea.

Daily Confirmed Cases (Raw)

	ARIMA	Bi-LSTM	Extended SEIRD	GAM	Holt-Winters	LightGBM	Time Series Poisson
**Null**	**8.366e+08**	**1.554e+09**	**1.003e+09**	8.683e+08	**8.034e+08**	**3.351e+08**	1.323e+09
**BA.5 rate**	8.381e+08	1.581e+09		4.550e+08		9.872e+08	1.125e+09
**BSR**	8.370e+08	1.664e+09		2.550e+08		4.517e+09	2.489e+09
**BSR+BA.5 rate**	8.369e+08	1.605e+09		**2.500e+08**		5.674e+08	**1.026e+09**

Daily Confirmed Cases (Smoothed)

	ARIMA	Bi-LSTM	Extended SEIRD	GAM	Holt-Winters	LightGBM	Time Series Poisson
**Null**	**2.606e+07**	1.545e+08	**1.727e+08**	6.490e+08	**2.182e+07**	5.415e+08	2.460e+07
**BA.5 rate**	2.619e+07	6.065e+07		2.344e+08		**2.542e+08**	2.346e+07
**BSR**	2.613e+07	4.914e+07		4.693e+07		5.412e+08	6.117e+08
**BSR+BA.5 rate**	2.620e+07	**3.803e+07**		**4.532e+07**		4.276e+09	**2.021e+07**

Daily confirmed deaths (Raw)

	ARIMA	Bi-LSTM	Extended SEIRD	GAM	Holt-Winters	LightGBM	Time Series Poisson
**Null**	**384.249**	**1328.574**	**1597.072**	2195.110	**1067.032**	1907.761	1310.874
**BA.5 rate**	408.029	1616.917		1033.810		2433.336	**911.200**
**BSR**	407.609	1333.622		477.061		**652.240**	1271.447
**BSR+BA.5 rate**	413.237	1418.474		**460.705**		2195.858	2072.723

Daily confirmed deaths (Smoothed)

	ARIMA	Bi-LSTM	Extended SEIRD	GAM	Holt-Winters	LightGBM	Time Series Poisson
**Null**	**17.537**	125.267	**569.533**	2023.982	**27.577**	156.601	**22.715**
**BA.5 rate**	17.692	229.494		693.629		165.885	565.107
**BSR**	17.594	**55.670**		56.806		**150.797**	204.741
**BSR+BA.5 rate**	17.593	240.841		**39.200**		168.204	196.779

ICU patients (Raw)

	ARIMA	Bi-LSTM	Extended SEIRD	GAM	Holt-Winters	LightGBM	Time Series Poisson
**Null**	212.121	1595.852		51464.926	**1054.216**	1737.357	957.226
**BA.5 rate**	221.743	2574.747		18472.768		1814.881	9774.497
**BSR**	**158.322**	3025.071		1347.703		2800.224	**849.874**
**BSR+BA.5 rate**	160.963	**1197.666**		**1134.643**		**1680.146**	850.083

ICU patients (Smoothed)

	ARIMA	Bi-LSTM	Extended SEIRD	GAM	Holt-Winters	LightGBM	Time Series Poisson
**Null**	333.477	381.372		49153.064	**125.126**	789.905	31733.766
**BA.5 rate**	**311.096**	495.050		18503.071		907.485	186.863
**BSR**	312.203	**352.898**		845.804		1246.678	76.459
**BSR+BA.5 rate**	312.102	1453.067		**798.998**		**663.373**	**75.951**

Table S9. The r^2 values for all covariate combinations for each single model for Korea.

Daily Confirmed Cases (Raw)

	ARIMA	Bi-LSTM	Extended SEIRD	GAM	Holt-Winters	LightGBM	Time Series Poisson
**Null**	**0.941**	**0.876**	**0.913**	0.925	**0.935**	**0.986**	0.886
**BA.5 rate**	0.941	0.874		0.961		0.971	0.906
**BSR**	0.940	0.872		0.978		0.781	0.791
**BSR+BA.5 rate**	0.940	0.875		**0.978**		0.971	**0.914**

Daily Confirmed Cases (Smoothed)

	ARIMA	Bi-LSTM	Extended SEIRD	GAM	Holt-Winters	LightGBM	Time Series Poisson
**Null**	**0.998**	0.989	**0.990**	0.939	**0.998**	0.995	0.998
**BA.5 rate**	0.998	0.997		0.978		**0.998**	0.998
**BSR**	0.998	**0.998**		0.996		0.993	0.945
**BSR+BA.5 rate**	0.998	0.997		**0.996**		0.944	**0.998**

Daily confirmed deaths (Raw)

	ARIMA	Bi-LSTM	Extended SEIRD	GAM	Holt-Winters	LightGBM	Time Series Poisson
**Null**	**0.965**	0.891	**0.877**	0.800	**0.909**	0.986	0.884
**BA.5 rate**	0.963	0.871		0.905		0.969	**0.919**
**BSR**	0.963	**0.892**		0.954		0.980	0.885
**BSR+BA.5 rate**	0.962	0.886		**0.956**		**0.989**	0.817

Daily confirmed deaths (Smoothed)

	ARIMA	Bi-LSTM	Extended SEIRD	GAM	Holt-Winters	LightGBM	Time Series Poisson
**Null**	**0.998**	0.993	**0.961**	0.800	**0.997**	0.999	**0.998**
**BA.5 rate**	0.998	0.978		0.933		**0.999**	0.945
**BSR**	0.998	**0.997**		0.994		0.999	0.980
**BSR+BA.5 rate**	0.998	0.987		**0.996**		0.999	0.981

ICU patients (Raw)

	ARIMA	Bi-LSTM	Extended SEIRD	GAM	Holt-Winters	LightGBM	Time Series Poisson
**Null**	0.998	0.990		0.589	**0.992**	0.993	0.993
**BA.5 rate**	0.998	0.979		0.855		0.995	0.926
**BSR**	**0.999**	0.985		0.989		0.992	**0.993**
**BSR+BA.5 rate**	0.999	**0.990**		**0.991**		**0.996**	0.993

ICU patients (Smoothed)

	ARIMA	Bi-LSTM	Extended SEIRD	GAM	Holt-Winters	LightGBM	Time Series Poisson
**Null**	0.997	**0.998**		0.614	**0.999**	0.997	0.780
**BA.5 rate**	**0.997**	0.997		0.857		**0.998**	0.999
**BSR**	0.997	0.998		0.993		0.998	0.999
**BSR+BA.5 rate**	0.997	0.989		**0.994**		0.998	**0.999**

Table S10. The selected error measures are used to calculate the weight in each WAE model.

Raw / Smoothed	Response variable	Error measures to calculate the weights
Raw	Daily Confirmed Cases	MSE_train
	Daily Confirmed Deaths	MSE_train
	Daily ICU Patients	MSE_train
Smoothed	Daily Confirmed Cases	MSE_train
	Daily Confirmed Deaths	MSE_train
	Daily ICU Patients	MAPE_train

Table S11. Comparison between the first-best and second-best models on test data for Korea.

Raw / Smoothed	Response variable	Error measure	First best model: Model name	First best model: Error value	Second best model: Model name	Second best model: Error value	Absolute difference of error values
Raw	Daily Confirmed Cases	WMAPE	**Stacking Ensemble (SVM)**	0.2353	GAM	0.2444	0.0091
		MAPE	GAM	26.5349	**Stacking Ensemble (SVM)**	27.4088	0.8739
		MSE	**Stacking Ensemble (SVM)**	9.38e+07	GAM	1.58e+08	6.40e+07
		RMSE	**Stacking Ensemble (SVM)**	9683.991	GAM	12562.66	2878.664
		r^2	**Stacking Ensemble (LR)**	0.936	LightGBM	0.9301	0.0059
	Daily Confirmed Deaths	WMAPE	**Stacking Ensemble (SVM)**	0.1177	**Weighted Average Ensemble**	0.119	0.0013
		MAPE	**Stacking Ensemble (SVM)**	16.9015	**Average Ensemble**	17.4184	0.5169
		MSE	**Stacking Ensemble (SVM)**	70.4556	**Average Ensemble**	78.6074	8.1518
		RMSE	**Stacking Ensemble (SVM)**	8.3938	**Average Ensemble**	8.8661	0.4723
		r^2	GAM	0.6844	Time Series Poisson	0.6486	0.0358
	Daily ICU Patients	WMAPE	**Stacking Ensemble (SVM)**	0.0193	Time Series Poisson	0.0218	0.0025
		MAPE	**Stacking Ensemble (SVM)**	1.9401	Time Series Poisson	2.2037	0.2636
		MSE	Holt-Winters	144.5502	**Stacking Ensemble (SVM)**	161.0596	16.5094
		RMSE	Holt-Winters	12.0229	**Stacking Ensemble (SVM)**	12.6909	0.668
		r^2	Holt-Winters	0.2257	**Average Ensemble**	0.0215	0.2043
Smoothed	Daily Confirmed Cases	WMAPE	Holt-Winters	0.0575	**Weighted Average Ensemble**	0.0598	0.0023
		MAPE	Holt-Winters	6.4849	**Average Ensemble**	6.618	0.1331
		MSE	**Average Ensemble**	1.32e+07	Holt-Winters	1.46e+07	1.48e+06
		RMSE	**Average Ensemble**	3626.812	Holt-Winters	3824.683	197.871
		r^2	ARIMA	0.9398	**Stacking Ensemble (LR)**	0.9273	0.0126
	Daily Confirmed Deaths	WMAPE	ARIMA	0.0583	**Stacking Ensemble (LR)**	0.0865	0.0282
		MAPE	ARIMA	5.7793	**Stacking Ensemble (LR)**	8.6046	2.8253
		MSE	ARIMA	11.3387	**Stacking Ensemble (LR)**	25.5007	14.162
		RMSE	ARIMA	3.3673	**Stacking Ensemble (LR)**	5.0498	1.6825
		r^2	Holt-Winters	0.0776	GAM	0.0334	0.0442
	Daily ICU Patients	WMAPE	**Weighted Average Ensemble**	0.013	ARIMA	0.0131	0.0001
		MAPE	**Weighted Average Ensemble**	1.3237	ARIMA	1.3239	0.0002
		MSE	ARIMA	71.0381	**Weighted Average Ensemble**	72.1149	1.0768
		RMSE	ARIMA	8.4284	**Weighted Average Ensemble**	8.492	0.0636
		r^2	Bi-LSTM	0.9678	Time Series Poisson	0.964	0.0038

Table S12. The error values for the best covariate combination for each model and the error methods for the USA.

	**Daily Confirmed Cases (Raw)**					**Daily Confirmed Cases (Smoothed)**				
	**MSE**	**RMSE**	**MAPE**	**WMAPE**	r^2	**MSE**	**RMSE**	**MAPE**	**WMAPE**	r^2
**ARIMA**	omicron [3.192e+10]	omicron [178664.217]	omicron [41.832]	omicron [0.339]	omicron [0.822]	omicron [4.211e+08]	omicron [20520.684]	omicron [6.002]	omicron [0.050]	omicron [0.995]
**Bi-LSTM**	omicron [2.028e+10]	omicron [142395.210]	omicron [50.974]	omicron [0.311]	omicron [0.929]	BSR-omicron [1.236e+09]	BSR-omicron [35150.010]	Null [17.598]	BSR-omicron [0.101]	BSR-omicron [0.998]
**GAM**	BSR-omicron [8.165e+09]	BSR-omicron [90361.097]	BSR-omicron [25.852]	BSR-omicron [0.202]	BSR-omicron [0.919]	BSR-omicron [1.382e+09]	BSR-omicron [37178.799]	BSR-omicron [11.671]	BSR-omicron [0.097]	BSR-omicron [0.998]
**LightGBM**	omicron [2.678e+09]	omicron [51748.062]	omicron [14.908]	omicron [0.117]	BSR [0.986]	BSR [2.634e+09]	BSR [51321.514]	BSR [6.819]	BSR [0.107]	BSR [0.999]
**time series Poisson**	BSR-omicron [2.633e+10]	BSR-omicron [162261.220]	BSR-omicron [50.121]	BSR-omicron [0.334]	BSR-omicron [0.936]	BSR-omicron [5.995e+09]	BSR-omicron [77426.229]	BSR-omicron [26.144]	BSR-omicron [0.172]	BSR-omicron [0.982]
	**Daily Confirmed Deaths (Raw)**					**Daily Confirmed Deaths (Smoothed)**				
	**MSE**	**RMSE**	**MAPE**	**WMAPE**	r^2	**MSE**	**RMSE**	**MAPE**	**WMAPE**	r^2
**ARIMA**	Null [5.504e+05]	Null [741.905]	omicron [44.805]	Null [0.329]	Null [0.781]	Null [2.416e+03]	Null [49.155]	Null [2.195]	Null [0.021]	Null [0.996]
**Bi-LSTM**	BSR [2.736e+05]	BSR [523.067]	BSR [41.524]	BSR [0.254]	BSR [0.895]	omicron [8.772e+03]	omicron [93.661]	omicron [4.431]	omicron [0.037]	omicron [0.992]
**GAM**	BSR-omicron [1.183e+05]	BSR-omicron [343.890]	BSR-omicron [25.132]	BSR-omicron [0.152]	BSR-omicron [0.910]	BSR-omicron [1.866e+04]	BSR-omicron [136.614]	BSR-omicron [7.664]	BSR-omicron [0.068]	BSR-omicron [0.985]
**LightGBM**	Null [5.503e+05]	Null [741.828]	Null [40.415]	Null [0.384]	omicron [0.986]	BSR [5.681e+03]	BSR [75.373]	BSR [2.704]	BSR [0.032]	BSR-omicron [0.997]
**time series Poisson**	BSR-omicron [3.031e+05]	BSR-omicron [550.581]	BSR [32.418]	omicron [0.226]	BSR-omicron [0.772]	BSR-omicron [1.461e+05]	BSR-omicron [382.205]	BSR-omicron [18.349]	BSR-omicron [0.168]	BSR-omicron [0.939]
	**ICU patients (Raw)**					**ICU patients (Smoothed)**				
	**MSE**	**RMSE**	**MAPE**	**WMAPE**	r^2	**MSE**	**RMSE**	**MAPE**	**WMAPE**	r^2
**ARIMA**	omicron [1.444e+05]	omicron [380.035]	BSR [1.758]	omicron [0.017]	omicron [0.995]	omicron [1.325e+05]	omicron [363.968]	omicron [1.626]	omicron [0.016]	omicron [0.995]
**Bi-LSTM**	BSR [2.385e+05]	BSR [488.396]	BSR [2.320]	BSR [0.024]	omicron [0.998]	omicron [2.539e+05]	omicron [503.924]	BSR-omicron [2.270]	omicron [0.022]	BSR-omicron [0.999]
**GAM**	BSR-omicron [3.678e+04]	BSR-omicron [191.779]	BSR-omicron [1.017]	BSR-omicron [0.009]	BSR-omicron [1.000]	BSR-omicron [3.522e+05]	BSR-omicron [593.450]	BSR-omicron [3.187]	BSR-omicron [0.028]	BSR-omicron [0.999]
**LightGBM**	BSR-omicron [3.956e+04]	BSR-omicron [198.886]	BSR-omicron [1.120]	BSR-omicron [0.011]	omicron [1.000]	omicron [1.147e+05]	omicron [338.727]	omicron [1.889]	omicron [0.019]	omicron [1.000]
**time series Poisson**	omicron [3.942e+06]	omicron [1985.379]	omicron [9.003]	omicron [0.087]	omicron [0.961]	BSR [3.411e+06]	BSR [1846.920]	BSR [8.124]	BSR [0.080]	BSR [0.942]

Table S13. Comparison between the first-best and second-best models on test data for the USA.

Raw / Smoothed	Response variable	Error measure	First best model		Second best model		Absolute difference of error values
			Model name	Error value	Model name	Error value	
Raw	Daily Confirmed Cases	WMAPE	GAM	0.2509	Time Series Poisson	0.2984	0.0475
		MAPE	GAM	32.1687	**Stacking Ensemble (SVM)**	32.7418	0.5731
		MSE	GAM	4.12E+08	Time Series Poisson	7.97E+08	3.85E+08
		RMSE	GAM	20297.8712	Time Series Poisson	28231.0583	7933.1872
		r^2	GAM	0.5017	Holt-Winters	0.2312	0.2704
	Daily Confirmed Deaths	WMAPE	ARIMA	0.1762	**Weighted Average Ensemble**	0.197	0.0208
		MAPE	ARIMA	28.3623	**Weighted Average Ensemble**	31.4959	3.1336
		MSE	**Average Ensemble**	3.00E+05	ARIMA	3.35E+05	34859.2255
		RMSE	**Average Ensemble**	547.5762	ARIMA	578.5317	30.9555
		r^2	Holt-Winters	0.7979	Time Series Poisson	0.7344	0.0635
	Daily ICU Patients	WMAPE	**Weighted Average Ensemble**	0.0067	GAM	0.0091	0.0024
		MAPE	**Weighted Average Ensemble**	0.7009	**Stacking Ensemble (SVM)**	0.8978	0.1969
		MSE	**Weighted Average Ensemble**	5115.0321	**Stacking Ensemble (SVM)**	8168.8659	3053.8338
		RMSE	**Weighted Average Ensemble**	71.5195	**Stacking Ensemble (SVM)**	90.3818	18.8623
		r^2	Holt-Winters	0.999	**Weighted Average Ensemble**	0.9988	0.0002
Smoothed	Daily Confirmed Cases	WMAPE	**Stacking Ensemble (SVM)**	0.048	**Weighted Average Ensemble**	0.0537	0.0057
		MAPE	**Stacking Ensemble (SVM)**	4.7483	**Average Ensemble**	5.2952	0.5469
		MSE	LightGBM	2.15E+07	**Weighted Average Ensemble**	2.23E+07	8.14E+05
		RMSE	LightGBM	4632.6628	**Weighted Average Ensemble**	4719.7452	87.0824
		r^2	Time Series Poisson	0.9198	Bi-LSTM	0.9045	0.0153
	Daily Confirmed Deaths	WMAPE	**Average Ensemble**	0.0701	Holt-Winters	0.0716	0.0015
		MAPE	**Average Ensemble**	7.0831	Holt-Winters	7.3011	0.2179
		MSE	**Average Ensemble**	18344.6078	Holt-Winters	23444.0032	5099.3954
		RMSE	**Average Ensemble**	135.4423	Holt-Winters	153.1143	17.6721
		r^2	Time Series Poisson	0.9803	**Weighted Average Ensemble**	0.7996	0.1807
	Daily ICU Patients	WMAPE	**Stacking Ensemble (SVM)**	0.0013	**Stacking Ensemble (LR)**	0.0019	0.0006
		MAPE	**Stacking Ensemble (SVM)**	0.1332	**Stacking Ensemble (LR)**	0.1947	0.0615
		MSE	**Stacking Ensemble (SVM)**	212.8753	**Stacking Ensemble (LR)**	439.8658	226.9905
		RMSE	**Stacking Ensemble (SVM)**	14.5902	**Stacking Ensemble (LR)**	20.973	6.3827
		r^2	**Weighted Average Ensemble**	0.9998	**Stacking Ensemble (SVM)**	0.9998	0

Table S14. Mean and variance of error measures for Korea analysis.

Raw / Smoothed	Response variable	Error measure	Mean		Variance	
			Ensemble	Individual	Ensemble	Individual
Raw	Daily Confirmed Cases	WMAPE	0.327	0.366	4.037.E-03	8.096.E-03
		MAPE	37.508	43.351	4.588.E+01	7.927.E+01
		MSE	1.90.E+08	3.03.E+08	4.153.E+15	1.019.E+16
		RMSE	1.36.E+04	1.72.E+04	6.854.E+06	9.453.E+06
		r^2	0.767	0.359	3.201.E-02	9.929.E-02
	Daily Confirmed Deaths	WMAPE	0.130	0.280	3.585.E-04	1.686.E-02
		MAPE	18.561	32.330	5.776.E+00	2.438.E+02
		MSE	85.384	297.965	3.505.E+02	8.899.E+04
		RMSE	9.202	16.025	9.505.E-01	4.803.E+01
		r^2	0.563	0.338	6.482.E-04	1.026.E-01
	Daily ICU Patients	WMAPE	0.024	0.034	1.084.E-05	4.297.E-04
		MAPE	2.396	3.422	1.139.E-01	4.382.E+00
		MSE	207.129	480.459	1.390.E+03	3.206.E+05
		RMSE	14.347	19.592	1.736.E+00	1.159.E+02
		r^2	0.014	0.039	2.898.E-05	8.352.E-03
Smoothed	Daily Confirmed Cases	WMAPE	0.077	0.085	4.306.E-04	6.636.E-04
		MAPE	8.433	9.155	5.847.E+00	6.793.E+00
		MSE	2.474.E+07	3.135.E+07	2.962.E+14	3.213.E+14
		RMSE	4781.966	5403.161	2.491.E+06	2.512.E+06
		r^2	0.798	0.771	7.514.E-03	1.003.E-02
	Daily Confirmed Deaths	WMAPE	0.109	0.118	3.515.E-04	1.555.E-03
		MAPE	10.849	11.719	3.490.E+00	1.550.E+01
		MSE	39.732	48.765	1.293.E+02	7.215.E+02
		RMSE	6.252	6.728	8.631.E-01	4.085.E+00
		r^2	0.018	0.027	1.660.E-04	6.240.E-04
	Daily ICU Patients	WMAPE	0.018	0.032	1.410.E-05	8.804.E-04
		MAPE	1.776	3.207	1.487.E-01	9.027.E+00
		MSE	129.924	586.978	3.334.E+03	1.164.E+06
		RMSE	11.191	18.705	6.253.E+00	2.845.E+02
		r^2	0.815	0.819	1.106.E-02	4.355.E-02

Table S15. Mean and variance of error measures for USA analysis.

Raw / Smoothed	Response variable	Error measure	Mean		Variance	
			**Ensemble**	**Individual**	**Ensemble**	**Individual**
**Raw**	**Daily Confirmed Cases**	WMAPE	0.335	0.494	5.025.E-05	5.182.E-02
		MAPE	39.329	65.416	3.578.E+01	1.496.E+03
		MSE	9.737.E+08	1.646.E+09	1.030.E+16	1.505.E+18
		RMSE	3.117.E+04	3.842.E+04	2.590.E+06	1.979.E+08
		r^2	0.141	0.160	2.402.E-04	2.883.E-02
	**Daily Confirmed Deaths**	WMAPE	0.233	0.312	1.181.E-03	1.864.E-02
		MAPE	35.069	42.864	7.960.E+00	1.405.E+02
		MSE	3.630.E+05	6.475.E+05	3.221.E+09	2.679.E+11
		RMSE	601.101	766.775	2.191.E+03	6.948.E+04
		r^2	0.653	0.584	3.285.E-03	6.812.E-02
	**Daily ICU Patients**	WMAPE	0.018	0.067	2.655.E-04	1.095.E-02
		MAPE	1.824	6.896	2.884.E+00	1.137.E+02
		MSE	4.205.E+04	1.036.E+06	4.093.E+09	5.585.E+12
		RMSE	166.037	606.103	1.931.E+04	8.022.E+05
		r^2	0.998	0.992	5.023.E-07	8.065.E-05
**Smoothed**	**Daily Confirmed Cases**	WMAPE	0.070	0.203	1.387.E-03	3.095.E-02
		MAPE	7.267	20.988	1.705.E+01	3.179.E+02
		MSE	4.720.E+07	4.013.E+08	1.756.E+15	4.085.E+17
		RMSE	6.459.E+03	1.616.E+04	7.306.E+06	1.634.E+08
		r^2	0.882	0.881	3.963.E-05	8.326.E-04
	**Daily Confirmed Deaths**	WMAPE	0.103	0.162	6.242.E-04	1.175.E-02
		MAPE	10.460	16.401	6.694.E+00	1.159.E+02
		MSE	4.566.E+04	1.386.E+05	4.918.E+08	2.795.E+10
		RMSE	208.264	322.189	3.046.E+03	4.056.E+04
		r^2	0.579	0.454	1.484.E-01	6.154.E-02
	**Daily ICU Patients**	WMAPE	0.021	0.068	6.655.E-04	9.446.E-03
		MAPE	2.125	6.975	6.903.E+00	9.577.E+01
		MSE	9.910.E+04	1.279.E+06	2.298.E+10	7.877.E+12
		RMSE	219.087	710.873	6.814.E+04	9.282.E+05
		r^2	1.000	0.998	8.868.E-08	8.493.E-06

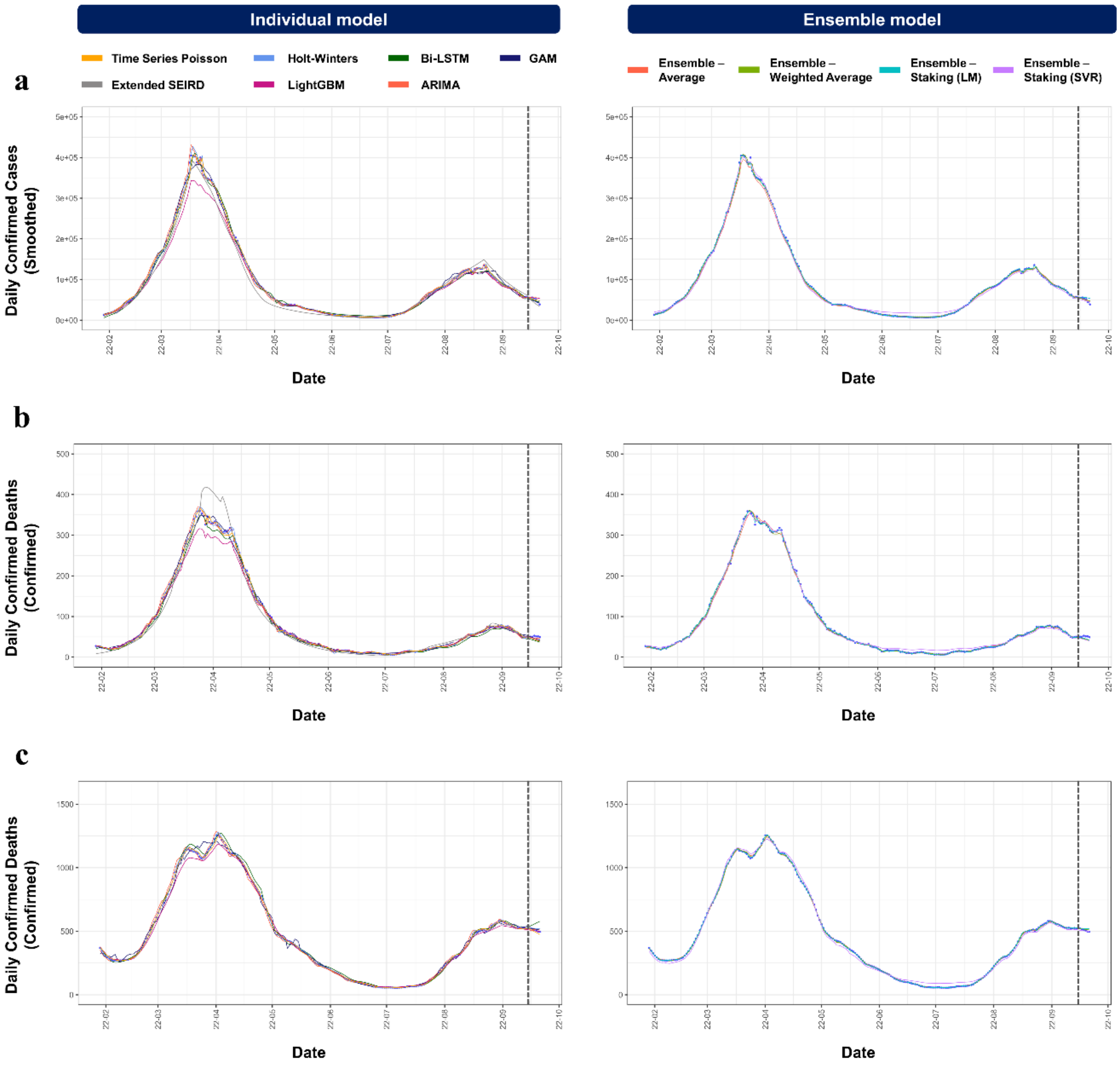

Figure S2. Plots showing the forecasting of daily confirmed cases, daily confirmed deaths and daily ICU patients for the seven individual models (ARIMA, GAM, LightGBM, Bi-LSTM, extended SIERD, Holt winter's and time series Poisson) and ensemble models with smoothed data for Korea. The right side of the vertical line marks the test period.

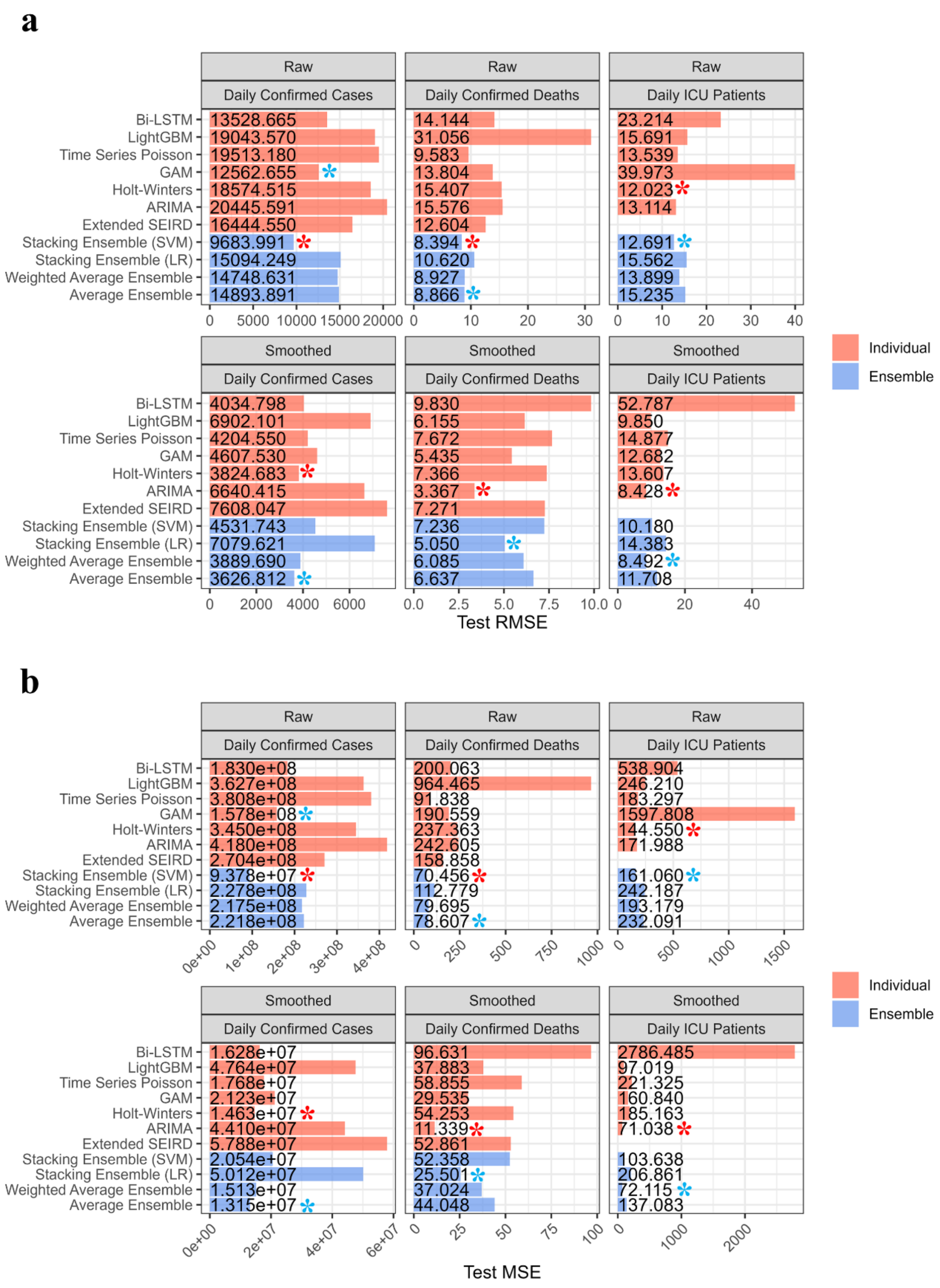

Figure S3. Summary of the performance of individual models and ensemble models using test data for Korea. (**a**) Performance using RMSE values. (**b**) Performance using MSE values. The horizontal bars represent the size of the error. The first best-performed models are marked with *, and the second best-performed models are marked with *.

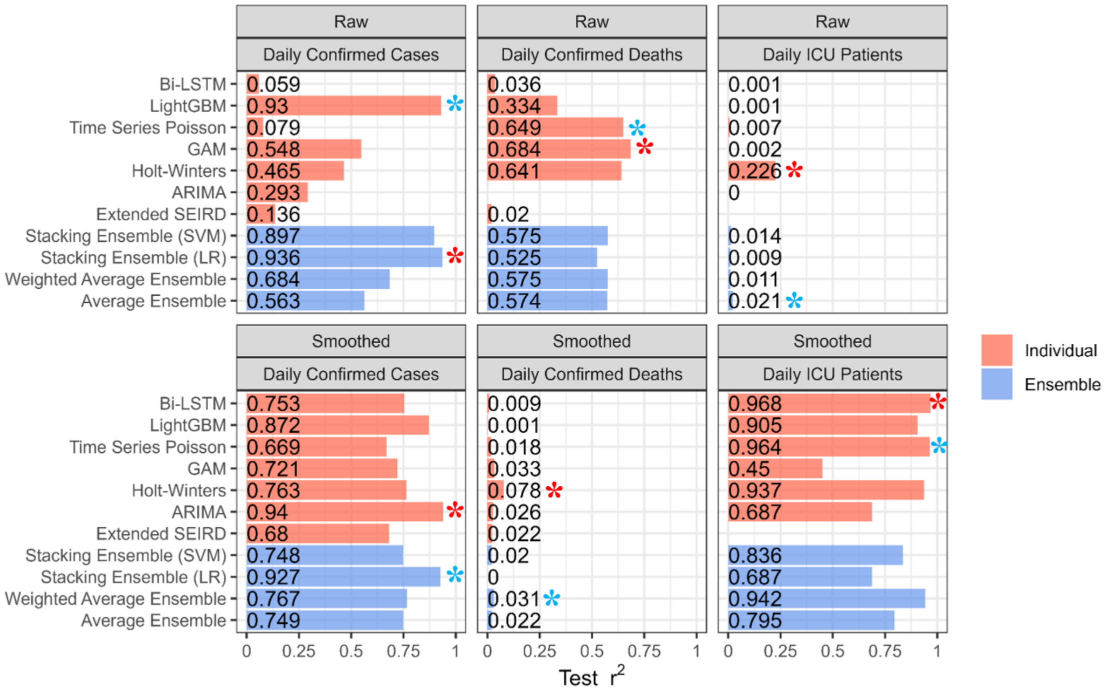

Figure S4. Summary of the performance of individual models and ensemble models for Korea, using test data using r^2. The horizontal bars represent the size of the r^2. The first best-performed models are marked with *, and the second best-performed models are marked with *.

a

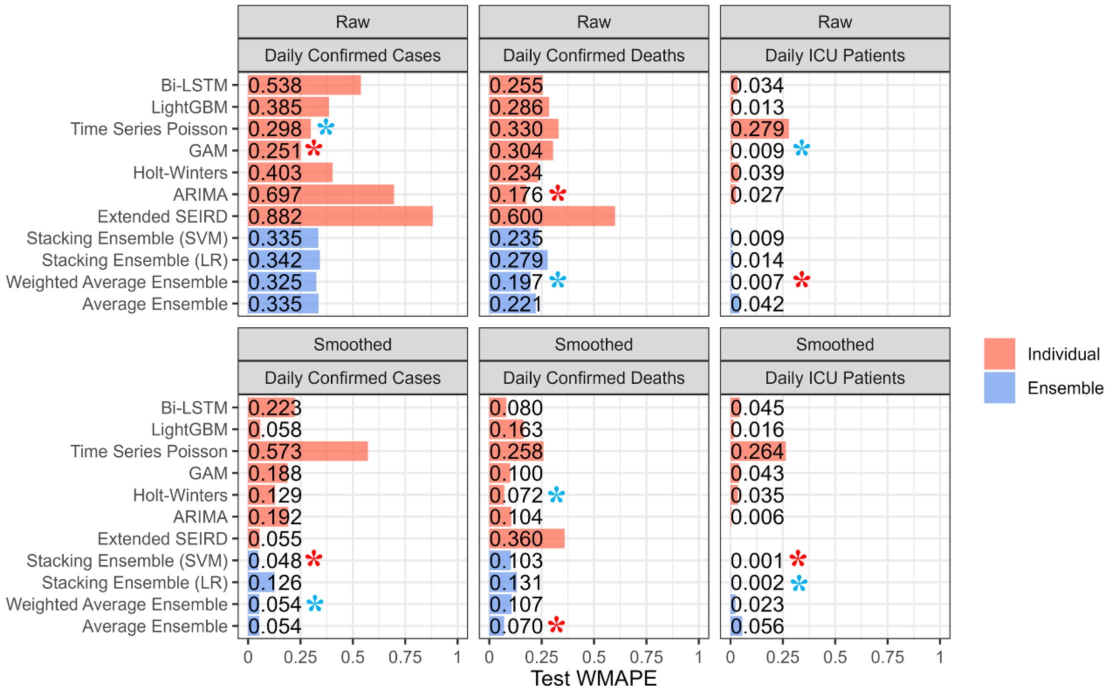

b

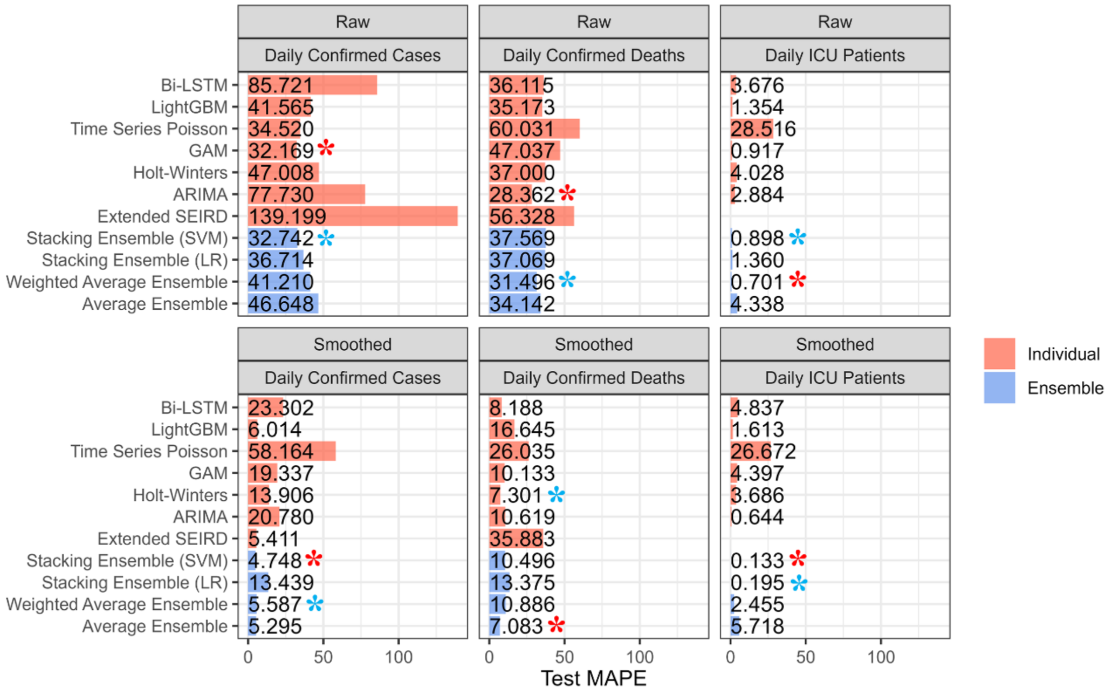

Figure S5. Summary of the performance of individual models and ensemble models using test data for USA. (**a**) Performance using WMAPE values. (**b**) Performance using MAPE values. The horizontal bars represent the size of the error. The first best-performed models are marked with *, and the second best-performed models are marked with *.

a

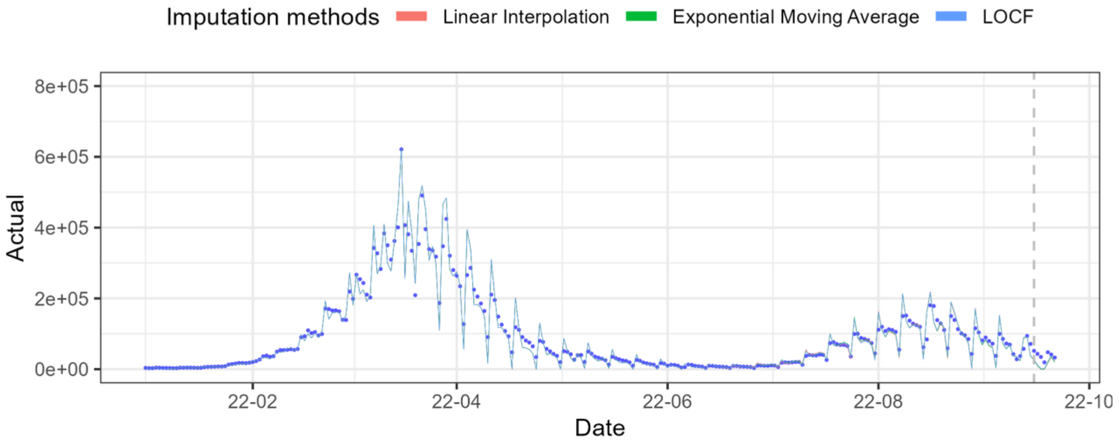

b

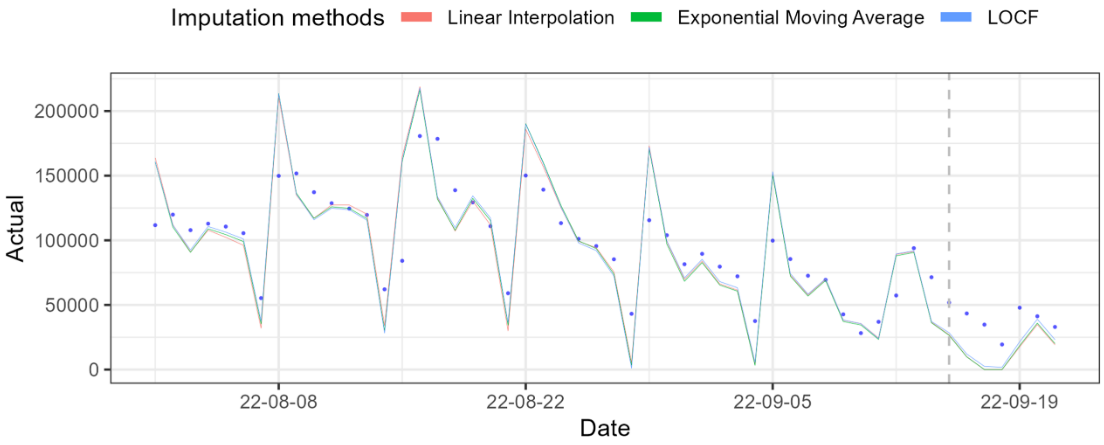

Figure S6. Small set analysis to assess the effect of different imputation methods on prediction results for (a) whole periods (2022-01-01 ~ 2022-09-22), and (b) recent periods (2022-08-01 ~ 2022-09-22). Three different methods were used to impute the BA.5 variant rate, and the ARIMA model using the BA.5 variant rate as covariates was fitted to predict raw daily confirmed cases.

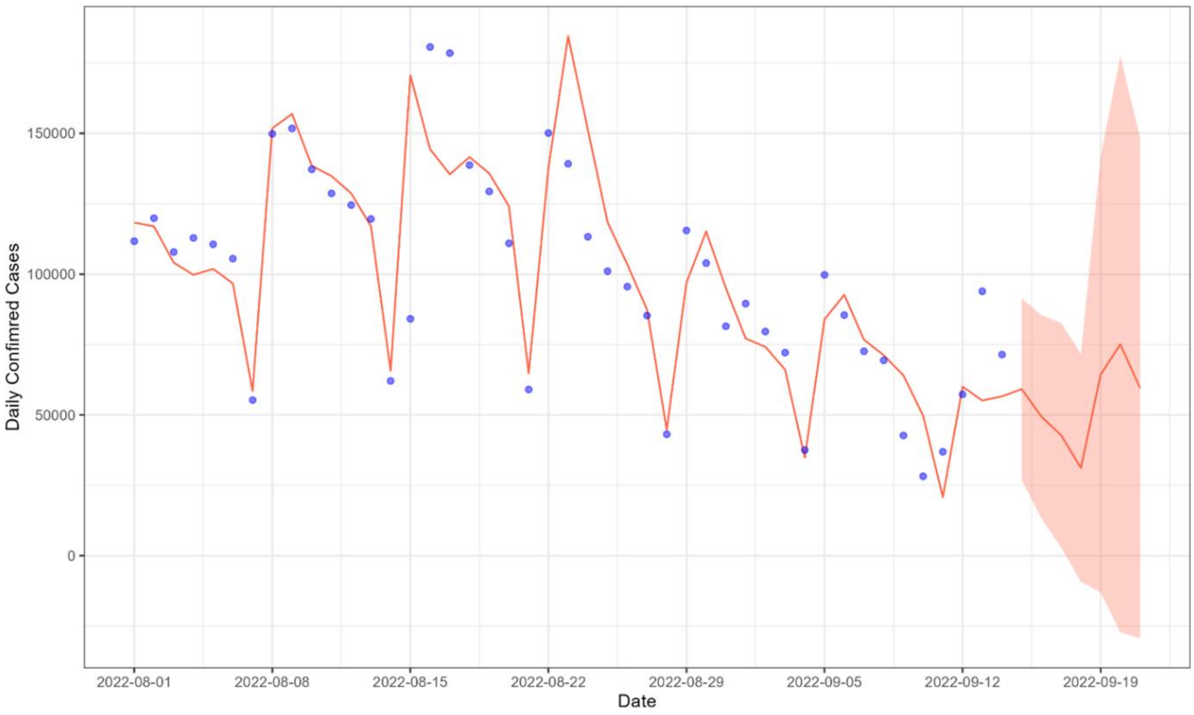

Figure S7. The confidence intervals (CIs) for the test period using the Holt-Winter's model

Supplementary Data Excel file can be downloaded at:

https://docs.google.com/spreadsheets/d/1WHpzlUKy4VAa6CPZJZ8Vgy6RNa9YhOF_/edit?usp=drive_link&ouid=107888743466298049125&rtpof=true&sd=true

References

1 Box GE, Jenkins GM, Reinsel G. Time series analysis: forecasting and control Holden-day San Francisco. BoxTime Series Analysis: Forecasting and Control Holden Day1970. 1970.

2 Yaffee RA, McGee M. An introduction to time series analysis and forecasting: with applications of SAS® and SPSS®: Elsevier; 2000.

3 Darapaneni N, Reddy D, Paduri AR, Acharya P, Nithin H, editors. Forecasting of COVID-19 in India using ARIMA model. 2020 11th IEEE Annual Ubiquitous Computing, Electronics & Mobile Communication Conference (UEMCON); 2020: IEEE.

4 Holt C. Forecasting seasonals and trends by exponentially weighted averages (ONR Memorandum No. 52). Carnegie Institute of Technology, Pittsburgh USA. 1957;10.

5 Winters PR. Forecasting sales by exponentially weighted moving averages. Management science. 1960;6:324-42.

6 Nelder JA, Wedderburn RW. Generalized Linear Models. J R Stat Soc Ser a-G. 1972;135:370-84.

7 Liboschik T, Fokianos K, Fried R. tscount: An R Package for Analysis of Count Time Series Following Generalized Linear Models. J Stat Softw. 2017;82.

8 Hastie T, Tibshirani R. Generalized Additive Models. Statistical Science. 1986;1:297-310.

9 Wood SN. Thin plate regression splines. J R Stat Soc B. 2003;65:95-114.

10 Wood SN. Stable and efficient multiple smoothing parameter estimation for generalized additive models. J Am Stat Assoc. 2004;99:673-86.

11 Wood SN. Fast stable restricted maximum likelihood and marginal likelihood estimation of semiparametric generalized linear models. J R Stat Soc B. 2011;73:3-36.

12 Menni C, May A, Polidori L, Louca P, Wolf J, Capdevila J, et al. COVID-19 vaccine waning and effectiveness and side-effects of boosters: a prospective community study from the ZOE COVID Study. Lancet Infect Dis. 2022;22:1002-10.

13 Albani V, Loria J, Massad E, Zubelli J. COVID-19 underreporting and its impact on vaccination strategies. Bmc Infect Dis. 2021;21.

14 Gumaei A, Al-Rakhami M, Al Rahhal MM, Albogamy FRH, Al Maghayreh E, AlSalman H. Prediction of COVID-19 Confirmed Cases Using Gradient Boosting Regression Method. Cmc-Comput Mater Con. 2021;66:315-29.

15 Ke G, Meng Q, Finley T, Wang T, Chen W, Ma W, et al. Lightgbm: A highly efficient gradient boosting decision tree. Advances in neural information processing systems. 2017;30.

16 Hochreiter S, Schmidhuber J. Long short-term memory. Neural computation. 1997;9:1735-80.

17 Graves A, Schmidhuber J. Framewise phoneme classification with bidirectional LSTM and other neural network architectures. Neural Networks. 2005;18:602-10.
